# ZBiotics ameliorates T2DM-induced histopathological damage in liver, kidney and adipose tissues by modulating the NOD-like receptor signaling in Wistar rats

**DOI:** 10.1186/s13098-025-01600-3

**Published:** 2025-02-04

**Authors:** Mohammed Esawie, Marwa Matboli, Mariam Sameh Bushra, Amany H. Hasanin, Eman Kamal Habib, Reham Hussein Mohamed, Hebatalla Said Ali

**Affiliations:** 1 Department of Medical Biochemistry and Molecular Biology, Faculty of Medicine, Ain-Shams University, Cairo, Egypt; 2 Clinical Pharmacology Department, Faculty of Medicine, Ain-Shams University, Cairo, Egypt; 3 Human Anatomy and Embryology Department, Faculty of Medicine, Ain-Shams University, Cairo, Egypt; 4 Molecular Biology Research Lab, Misr International University, Cairo, Egypt; 5 Human Anatomy and Embryology Department, Faculty of Medecine, Galala University, Suez, Egypt

**Keywords:** Type 2 diabetes mellitus, ZBiotics, TNF-alpha, LC3B, STING-NOD

## Abstract

Probiotics serve as promising candidates in type 2 diabetes mellitus (T2DM) therapy. Not only they presumably reduce the T2DM prevalence, but also keep down its complications. In the present study, we explored the beneficial impact of ZBiotics, an engineered probiotic, on T2DM Wistar rats. In silico analysis was performed to construct a genetic-epigenetic network linked to STING-NOD pathway and autophagy signaling. Then, 30 Wistar rats were divided into 5 groups (each n = 6); normal group, diabetic model, *B. subtilis*, and ZBiotics treated rats at high and low doses. Experimental procedures were carried out including biochemical and histopathologic analyses. Samples were extracted from rats’ blood, liver, kidney and adipose tissues. At the molecular aspect, the molecular players, chosen by the in silico analysis, were assessed using 2^–ΔΔCt^ to estimate their relative quantification. With immunohistochemistry, TNF-alpha and LC3B were assessed as reflectors for inflammation and autophagy respectively. ZBiotics was reported to ameliorate the T2DM-induced histological damage. Besides, it downregulated TNF-alpha and upregulated LC3B expression levels. At the biochemical aspect, ZBiotics corrected LDL-c and improved serum creatinine and CK-MB levels. Inflammation relevant genes have been downregulated regarding CHUK, NFKB1 and miR-611. Therefore, ZBiotics is speculated to operate by modulating the genetic-epigenetic network linked to inflammatory cGAS-STING and autophagy signaling. ZBiotics is recommended for clinical trials as a separate candidate or as an adjuvant to the conventional T2DM therapy.

**Supplementary Information:**

The online version contains supplementary material available at 10.1186/s13098-025-01600-3.

## Introduction

Diabetes Mellitus is a chronic status of hyperglycemia which, over time, inflicts serious damage on blood vessels, eyes, kidneys and nerves. The prevalence of diabetes mellitus type 2 has been growing steadily over the last decades. In 2021, the International Diabetes Federation has reported a global prevalence of 10.5% among adult population ranging from 20 to 79 years [1]. It’s associated with a surge in medical expenses and mortalities. Although the majority of people with diabetes mellitus type 2 are living in developing countries, diabetes mellitus health and economic burden affect all countries of all income levels [2].

Diabetes mellitus type 2 develops with a resistance to insulin action. This could be principally attributed to the intracellular lipogenesis which intervenes with the normal insulin signaling and releases a variety of adipokines and cytokines [3, 4]. The most important of which is TNF-alpha that not only modulates the migration and mitogenesis processes of vascular endothelial cells [5], but also helps the transcytosis of LDL-c into the subendothelial regions of diabetic blood vessels [6].

A solid evidence underscores the role of inflammation in insulin resistance and diabetes mellitus development [7, 8]. Herein, we entailed two major mechanisms evidently proven to trigger inflammation systematically with diabetes mellitus type 2. The first mechanism is the hyperglycemia induced oxidative stress that overcomes the cellular capability to contain it through autophagy, which is assessed by the estimation of LC3 proteins and is reported to be deficient in diabetes [9]. The second mechanism is gut dysbiosis and the horizontal gut leakage that facilitate the intravascular displacement of gram-negative bacteria associated lipopolysaccharides (LPS) and other microbial DNA fragments. Both biochemical structures foster NOD-like receptors; LPSs trigger them directly and microbial DNAs make use of cGAS-STING as triggering mediators [10]. STING is a transmembrane protein encoded by TMEM173 which not only activates JAK/ STAT pathway and produces more of INF [11], but also modulates oxidative stress operators, autophagy proteins [12] and facilitates diabetes mellitus development [8]. CHUK is one of the relevant operators to NOD-like receptors. It can inhibit Kappa Beta kinases to release the inhibitory effect they induce over NF-KB1, thus activating NF-KB1 at last [13].

In the present study, we utilized rat diabetic models to demonstrate the pathological outcomes of such mechanisms; histologically in liver, kidney and adipose tissues, and biochemically; by estimation of lipid profile, creatinine, CK-MB and other diabetes associated parameters. Molecularly, we assessed Nuclear factor kappa B subunit 1 (NFKB1) and Homo sapiens conserved helix-loop-helix ubiquitous kinase (CHUK) as both genes are eminent in the context of T2DM induced inflammation [14]. Also, miR-611 and lnc-RP4-60,503.4 were estimated due to their connection with NFKB1 and CHUK. This was suggested by thoroughly searching bioinformatic databases.

There is a global agreement on halting the rise in diabetes mellitus type 2 prevalence and preventing the associated dreadful complications. The problem of the conventional therapy is that it is not suitable for all cases as Thiazolidinediones that lead to liver toxicity, Metformin that is not suitable for renal insufficiency patients or patients with high risk to acidosis such as chronic pulmonary disease and chronic heart failure patients, alpha glucosidase inhibitors that’s have a lot of abdominal side effects as diarrhea, flatulence and abdominal pain, sulphonyl urea may also lead to hypoglycemia and weight gain. Therefore, our current study aims to search for novel therapeutic strategies for modulating inflammation with least side effects [15]. Besides the conventional therapy, probiotics has been reported to be promising candidates in diabetes mellitus therapy. Probiotics are defined as “live microorganisms that, when administrated in adequate amounts, confer a health benefit on the host” [16]. Food and beverages can be fortified with them to overcome diabetes treatment non-compliance [17]. However, probiotics effects in the domain of T2DM are general regarding the molecular targets and so, it would be more clinically beneficial if they were genetically engineered to acquire certain enzymes that would potentiate their original efficacy [18]. ZBiotics^®^ is the world’s first genetically engineered probiotic; *B. subtilis* spores were lyophilized and inoculated with acetaldehyde dehydrogenase gene derived from C. necator. Therefore, it’s purposefully used to limit hangover symptoms with alcohol drinking [19]. Acetaldehyde dehydrogenase could scavenge oxidative stress operators [20] which, if accumulated in large amounts, may lead to diabetes mellitus development [7, 21].

Also, Aldehyde dehydrogenase 2 (ALDH2) polymorphism has been reported as a risk factor for type 2 diabetes mellitus (T2DM) and is associated with liver insulin resistance [22]. Previous studies suggested that ALDH2 dysfunction may contribute to a variety of human diseases including cardiovascular diseases, diabetes, neurodegenerative diseases, stroke, and cancer [23]. Preclinical studies in rats also suggest that ALDH2 activity may affect diabetic pathology. Furthermore, in transgenic mice, overexpression of ALDH2 has been shown to be protective against streptozotocin-induced diabetic cardiomyopathy [24].

Therefore, the present study speculated that ZBiotics^®^ (modified *B. subtilis* strain that constitutively expressed with acetaldehyde dehydrogenase as a common oxidoreductase and one of the aldehyde dehydrogenases) that breaks down acetaldehyde would be a de novo therapeutic agent for ameliorating T2DM complications by modulating autophagy and manipulating oxidative stress mediators. This, in turn, could alleviate the degenerative diabetic changes in liver and kidney tissues as well as the visceral fat accumulation. Backed up with the histopathological outcome, it showcased ZBiotics^®^ impact on the diabetes affected biochemical parameters and the relative quantification of CHUK, NFKB1, miR-611 and lnc-RP4-60,503.4 as molecular players.

## Methods

### Bioinformatic analysis to identify genes and epigenetic modulators

Firstly, KEGG map (https://www.genome.jp/kegg/) was used to select a pathway related to insulin resistance and type 2 DM. Secondly, Bioinformatics was performed to find potential candidate genes related to T2DM, insulin resistance and NOD signaling pathway from earlier microarray research available in public databases; National Center of Biotechnology Information GEO (https://www.ncbi.nlm.nih.gov/geo/, available May 2022) and p value ≤ 0.05 was considered statistically significant. As a result, GSE78721 and GSE104948 datasets were selected as it contained adequate sample size with T2DM and control samples (details in supplementary Tables 1).

Then, the microarray data was analyzed using the online database tool GEO2R (https://www.ncbi.nlm.nih.gov/geo/geo2r/, accessed in May 2022) to detect genes with differential expressions. The criteria for screening used in this study were FDR < 0.05 and adj p value < 0.05. Probes without identified gene symbols were removed, and all the differentially expressed genes (DEGs) were then displayed through volcano graphs for better visualization.

The selected molecular players were filtered according to their high-ranking score, a strong link to insulin resistance signaling, type 2 diabetes mellitus, and the NOD pathway, their expression in the tissue of interest, kidney, liver and adipose tissue. Based on these criteria, CHUK and NFKB1 genes were selected.

Thirdly, Gene ontology (GO) enrichment of CHUK and NFKB1 mRNAs was performed using Enrichr (http://amp.pharm.mssm.edu/Enrichr) with p < 0.05 set as the cutoff criterion that verified its correlation to positive regulation of interferon production and kappa beta phosphorylation.

Fourthly, Protein atlas database (https://www.proteinatlas.org/) was employed to confirm the expression of the selected molecular players in target tissues, kidney, liver, adipose tissue and blood. Then, STRING online library (https://string-db.org/) was employed to confirm the protein–protein network regarding the selected elements. TNF-a and LC3B were linked at high confidence.

**Fig. 1:**
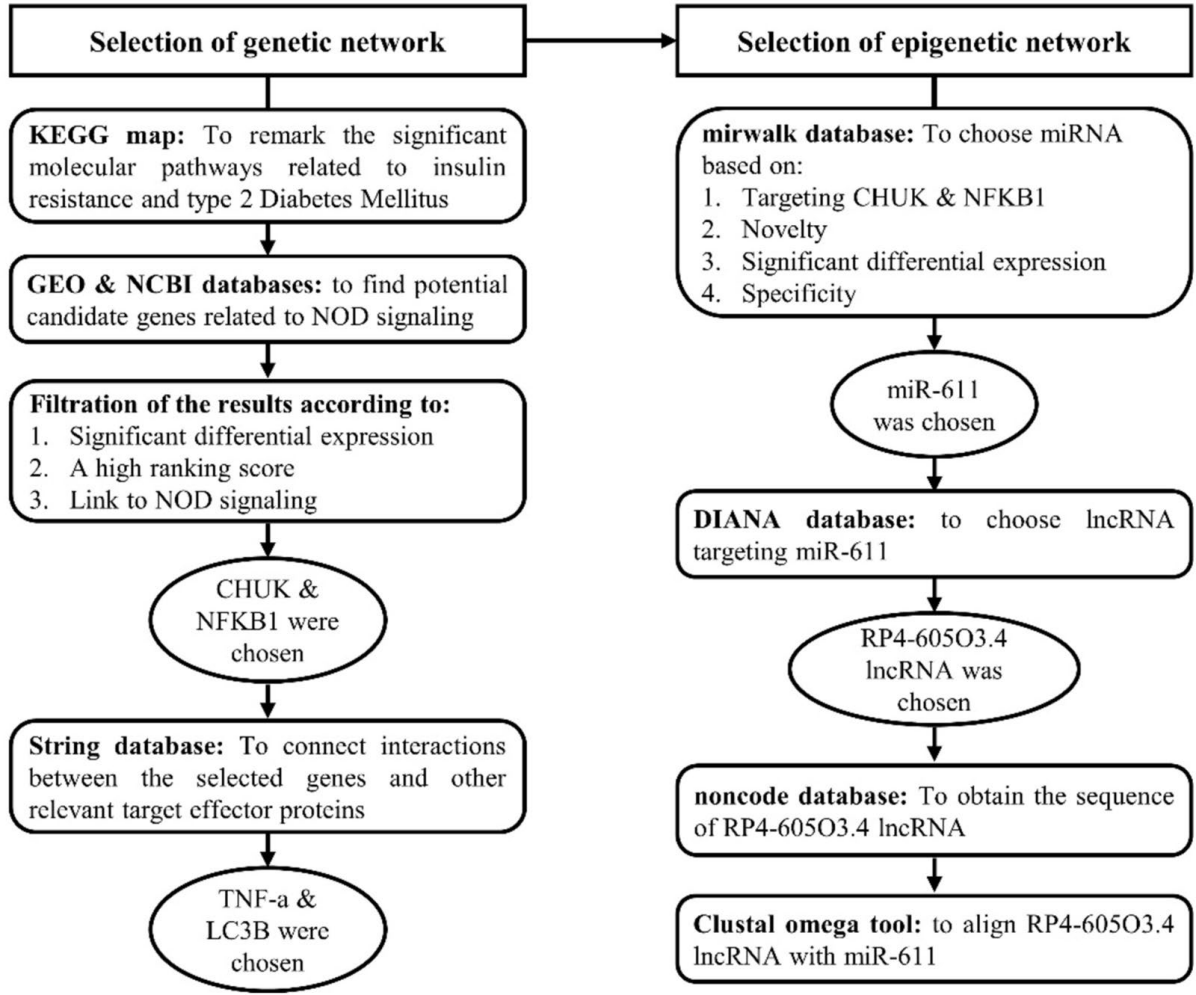
Workflow of bioinformatics steps

Fifthly, we selected the epigenetic regulatory elements of the extracted genes through epigenetic libraries such as mirwalk (http://mirwalk.umm.uni-heidelberg.de/), DIANA tools (http://diana.imis.athena-innovation.gr/DianaTools/index.php) and non-code (http://www.noncode.org/) databases. We selected miR-611 which can interact with both mRNAs selected before (score > 0.9 at CDS binding sites), also, pathway enrichment analysis showed its relation to inflammation. Additional 2 GEO series verified the expression of miR-611 in mice (GSE151334 and GSE111864). Finally, RP4-605O3.4 was found to interact with the selected miR-611 and that was confirmed by The European Bioinformatics Institute’s Clustal Omega program based on their high complementarity rate. Therefore, we have constructed the regulatory panel as CHUK and NFKB1 that can interact with miR_611 and lnc-RP4-605O3.4 (Fig. 1) and supplementary material (Figures S1–10).

### Chemicals

Streptozotocin and sodium citrate buffer were purchased from Sigma Chemical Co (St. Louis, Mo, USA) and ZBiotics^®^ was purchased from ZBiotics Co (Montgomery St, San Francisco, CA 94104, USA).

### Animals

50 Male Wistar rats of 140–185 g at the age of nearly two months were supplied by the Holding Company for Biological Products and Vaccines based in Giza, Egypt. They were let to adapt the laboratory conditions for one week with accessible water and food sources before diabetes induction. All animal protocols were conducted by the National Institutes of Health guide for the care and use of Laboratory Animals (8th edition, 2011) and were approved by the animal ethics committee for Ain-Shams University, Faculty of Medicine (Approval code: FWA 000017585).

### Induction of type 2 diabetes mellitus

**Fig. 2:**
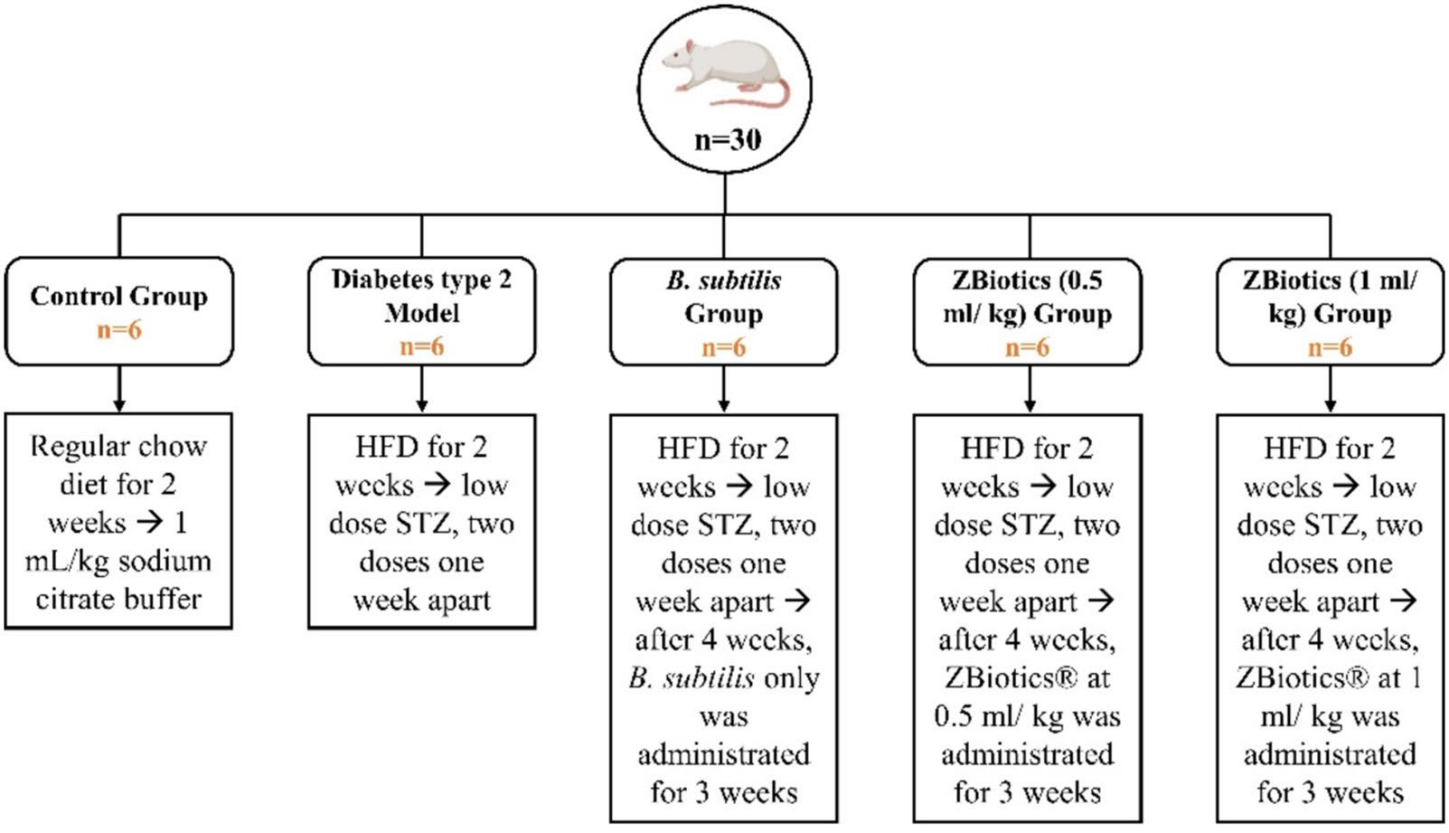
Workflow chart showing the experimental design of animal groups. *HFD* High Fat Diet, *STZ* Streptozotocin

Type 2 diabetes models were induced by feeding rats a high fat diet (HFD) (22% fat, 48% carbohydrates and 20% protein) for 2 weeks [25], followed by two intraperitoneal (i.p.) administration of low doses streptozotocin (STZ) with one week apart to cause pancreatic damage by DNA alkylation [26]. STZ was at a dosage of 30 mg/kg and was dissolved in citrate buffer of pH 4.5 in a volume of 1 mL/kg [21]. Instead of STZ, normal control models were treated by blank; 1 mL/kg sodium citrate buffer. After one week of the last STZ injection, collection of blood samples was carried out from tail veins, and then blood glucose was estimated with a glucometer (Accu-check, Roche Diagnostics, Risch-Rotkreuz, Switzerland). When non-fasting blood glucose levels were ≥ 200 mg/dl (11.1 mmol/ L), rats were considered diabetic [27]. The rats were allowed to continue to feed on their respective diets until the end of the study (Fig. 2).

### Experimental design

The animals under study were equally divided into 5 groups as follows; control group (n = 6): received regular chow diet (5% fat, 53% carbohydrates and 23% protein), STZ/HFD induced diabetic control group (n = 6): Each rat received HFD and intraperitoneal STZ with DMSO to facilitate its effect, quality group (n = 6) in which rats received only *B. subtilis* as a standard to ZBiotics^®^ effect. *B. subtilis* strains were grown at a shaker incubator; 30 °C, 220 rpm overnight in Lysogeny Broth (LB) medium with or without 5 μg/ml chloramphenicol. Overnight bacterial culture was diluted 10 times followed by 37 °C/ 220 rpm culturing. Evaluation of colony forming units (CFU) was assessed with spectrophotometric readings at OD600; target reading: between 0.6 and 0.8. For in vivo studies, bacteria were stored in formulation buffer (2.28 g/L KH2PO4, 14.5 g/L K2HPO4, 15% glycerol, pH 7.5) at 1011 CFU/ml at − 80 °C, equivalent CFUs to ZBiotics^®^ were administrated. Other two Diabetic + ZBiotics^®^ treated groups were designed (n = 6 per each dose group): 4 weeks after the last STZ injection, ZBiotics^®^ was administrated for 3 weeks at two doses: 0.5 ml/kg and 1 ml/kg. For the calculation of dosages, we performed a bacterial count assessment of ZBiotics^®^ using the standard plate count technique. This involved quantifying colonies to determine colony-forming units (CFU). Following this, ZBiotics^®^ dosage was determined based on prior research advocating a dosage of 10^9 CFU/kg, considered optimal for most probiotic strains. Consequently, in our current study, two dosages were administered: approximately 1 ml/kg (equivalent to 20 mg Zbiotic/mL) and 0.5 ml/kg (equivalent to 10 mg Zbiotic/0.5 mL). ZBiotics^®^ was administered orally through gastric gavage [19]. When the experiment was performed, blood samples were collected under ether anesthesia from retro-orbital vein. Then, rats were exterminated by cervical dislocation. Liver, kidney as well as adipose tissues were collected from all groups and fixed in 10% formalin for histopathological assessment (Fig. 2).

### Sampling and biochemical analysis

Blood samples were collected from rats’ optical vein before extermination. Then, they were centrifugated at 2000×*g* for 10 min at 4 °C and sera were collected. Commercial ELISAs kits were utilized for the estimation of cholesterol, triglycerides, HDL, LDL, urea nitrogen, serum creatinine, fasting blood sugar, CK-MB and troponin according to the manufacturers’ guidelines (RayBiotech, USA). Urine samples were collected the day before the treatment end. Urine albumin and creatinine levels were estimated utilizing colorimetric kits (RayBiotech, USA) as regards the supplier instructions.

### Total RNA extraction from liver, kidney and adipose tissue samples

Total RNA was isolated from the liver, kidney and adipose tissue samples utilizing the miRNeasy kit (Qiagen, Hilden, Germany). Using the DeNovix DS-11 microvolume spectrophotometer (Wilmington, USA), RNA purity and concentration were determined; the sample was read at 260 nm for RNA detection and at 280 nm for protein detection using the spectrophotometer. Based on 40 µg RNA/ ml is equivalent to 1 absorbance, the concentration of RNA in a sample (μg/ml) = sample absorbance at 260 nm × 40/1 × dilution factor. Consequently, the concentration of RNA in a sample is calculated by cross multiplication considering the dilution factor. The samples were considered with high RNA quality when the RNA: Protein ratio (260:280 ratios) was more than 1.8. Then, all RNA samples were stored at – 80 °C until PCR experiments. Real-time two-step RT-PCR was carried out on the extracted RNA by miRCURY LNA RT kit (Cat no. 339340; Qiagen, Hilden, Germany).

### Quantitative real-time polymerase chain reaction (qPCR)

The relative expression of NFKBI and CHUK mRNAs was measured by means of QuantiTect SYBR Green PCR Kit (Cat no. 204143; Qiagen, Hilden, Germany) with specific primers for Rn-CHUK_2_SG QuantiTect Primer Assay (NM_001107588)(Gene Globe ID: QT00382494) and RN_NFKB1_2_SG QuantiTect Primer Assay (NM_001276711)(Gene Globe ID: QT01577975). Relative miRNA expression levels for miR_611 were analyzed by miRCURY SYBR Green PCR Kit (Cat no. 339320; Qiagen, Hilden, Germany). Relative expression levels for lnc-RP4-605O3.4 were analyzed by QuantiNova Probe PCR Kit (Cat. no. 208252; Qiagen, Hilden, Germany) and QuantiNova LNA Probe PCR Assay for RP4-605O3.4 (ENST00000548468). GADPH was utilized as an internal control for the chosen mRNAs and lncRNA, and U6 was utilized as housekeeping genes for miRNA experiments. All samples were analyzed with the Applied Biosystems Tm 7500 Real-Time PCR system (Foster City, California, United States). Applied Biosystems 7500 software v2.3 was utilized for the estimation of the threshold cycle (Ct). The Ct values over 35 were regarded as negative. The amplicons’ specificities for the SYBR Green-based PCR amplification were confirmed by melting curve analysis. In this study, appropriate standardization strategies were carried out to recognize any experimental error introduced at any stage during the extraction and processing of the RNA according to MIQE guidelines, clarified in Supplementary materials, Table (S1).

### Histopathological examination

Liver, kidney and adipose tissue samples were dissected, fixed in 10% neutral buffered formalin for 72 h. Samples were processed in serial grades of ethanol, cleared in Xylene, and then embedded in Paraplast tissue embedding media. 5μ-thick tissue sections were cut by rotatory microtome and mounted on glass slides for the demonstration of hepatic lobules. Tissue sections were stained by Hematoxylin and Eosin as a standard stain for general microscopic examination by experienced histologist in a blinded manner. All standard procedures for samples fixation and staining were carried out according to Culling, C.F.A. 2013 [28].

### Immunohistochemistry assessment of LC3B and TNF-alpha

5 micron-thick paraffin embedded tissue sections were prepared for immune-histochemical assessment according to the manufacturer’s protocol. Deparaffinized retrieved tissue sections were treated by 0.3% H2O2 for 20 min. Liver and kidney samples were incubated with Anti TNF alpha (NBP1-19532—Novus Biologicals—1:100) and Anti LC3B (NB100-2220—Novus Biologicals—1:100) overnight at 4 °C. Tissue sections were washed out by PBS, followed by incubation with secondary antibody HRP Envision kit (DAKO) for 20 min. Then, washed out and incubated with diaminobenzidine (DAB) for 15 min. After that, they were washed by PBS, counter-stained with hematoxylin, dehydrated and cleared in xylene, then cover slipped for microscopic examination. According to Elsayed, 2022, at least 6 non-overlapping fields were randomly selected and scanned from tissue section of each renal sample as well as hepatic tissue samples for the determination of area percentage of immunohistochemical expression levels of TNF alpha and LC3B in immunohistochemically stained sections [29].

### Morphometric analysis of adipocytes

For adipocytes’ analysis, at least 6 non-overlapping fields were randomly selected and scanned from each tissue section for the determination of cell mean diameters. All light microscopic examination and data were obtained by using Leica Application module for histological analysis attached to Full HD microscopic imaging system (Leica Microsystems GmbH, Germany).

### Data analysis and statistics

The sample size was obtained utilizing GraphPadStatMate, software program version 1.01 (1998) (Inc., CA, USA). It was calculated for each variable, and the largest one was taken. The estimated sample size for each group was 6 rats. Results values were described as mean ± SD. The statistical analysis was done by means of GraphPad Prism software, version 10.2.1 (2024) (Inc., CA, USA). The statistical difference of the groups was calculated by means of analysis of variance (ANOVA), which was accompanied by post hoc “Turkey’s Multiple Comparison Test” to compare among more than two groups. Statistically significant were the p values (< 0.05). Using 2-DCt method, fold change in expression by RT-PCR was determined, where delta Ct = Ct gene of interest- Ct endogenous control. For statistical analyses as well as representation, 2-DCt values were log-transformed.

## Results

### Light microscopic examination (H&E)

**Fig. 3:**
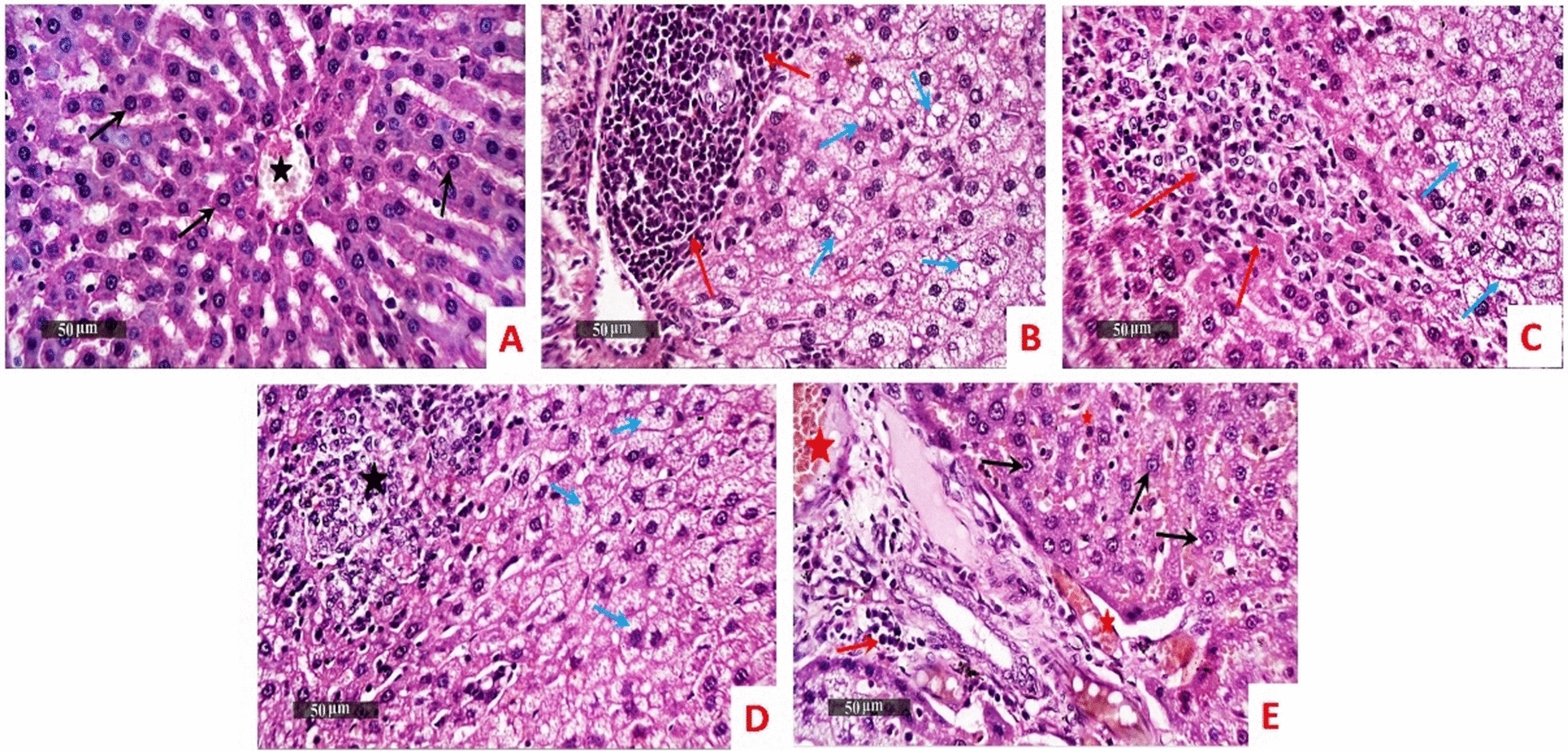
The effect of diabetic settings and ZBiotics use on liver tissues using HE (Magnifications: 50 µm). **A** Normal group **B** Diabetic model **C** *B. subtilis* **D** ZBiotics 0.5 ml/ kg **E** ZBiotics 1 ml/kg. Microvesicular steatosis alternated with vacuolar changes (blue arrow), inflammatory cells infiltrates (red arrow), focal hepatocellular necrosis (black star), congested hepatic vasculatures (red star), intact hepatocytes (black arrow)

Normal control demonstrated normal histological features of rat liver parenchyma. However, model samples showed diffuse hepatocellular microvesicular steatosis alternated with vacuolar changes allover hepatic lobules accompanied with moderate records of periportal mononuclear inflammatory cells infiltrates and focal figures of hepatocellular necrosis replaced with inflammatory cells. *B. subtilis* treated samples almost showed the same histopathological changes. With ZBiotics, the dosage 0.5 ml treated samples showed wide areas of vacuolar degenerative changes of most hepatic lobules with focal hepatocellular necrosis and congested hepatic vasculatures. Moreover, ZBiotics 1 ml treated samples demonstrated significant hepatocellular protective efficacy demonstrated with normal histological features. However, persistent dilatation of hepatic vasculatures was showed as well as congested hepatic sinusoids were showed with mild periportal inflammatory cells infiltrates (Fig. 3).

**Fig. 4:**
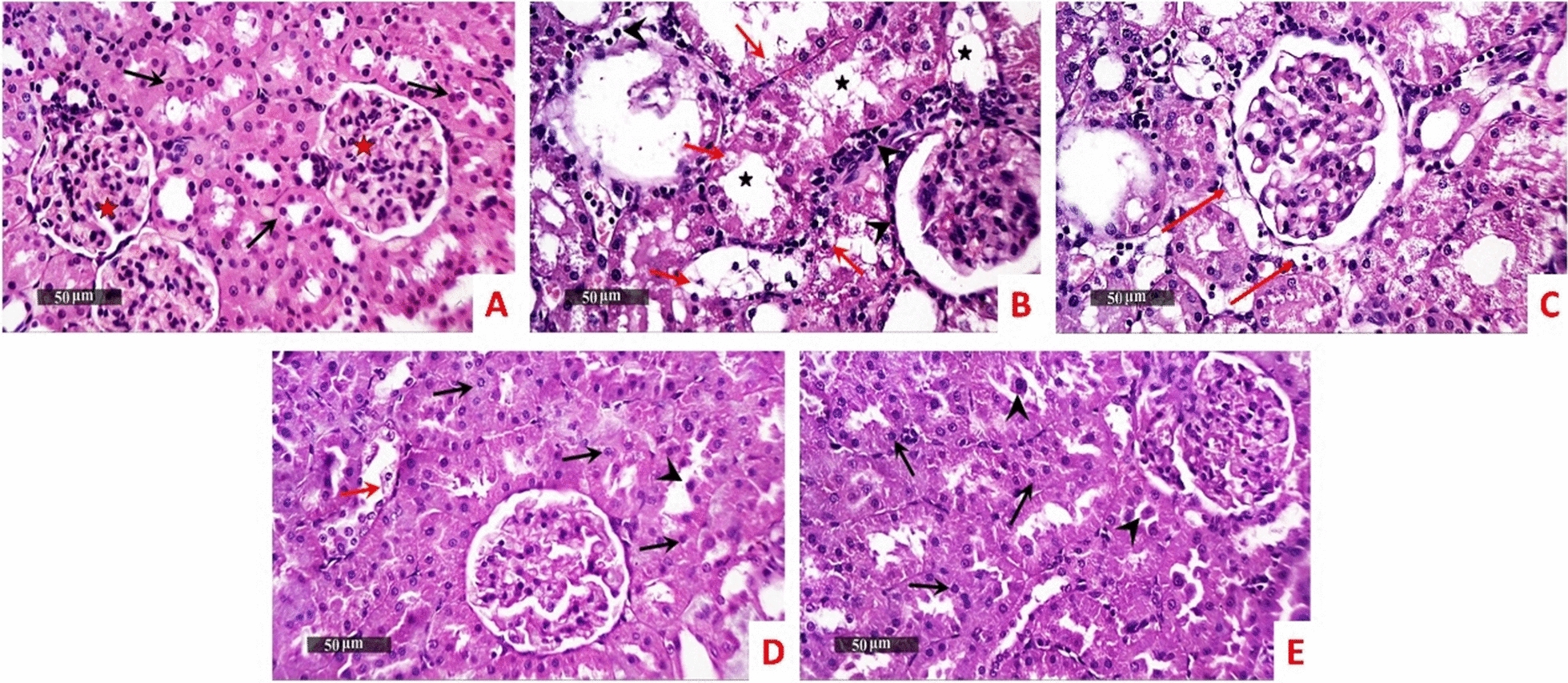
The effect of diabetic settings and ZBiotics use on kidney tissues using HE (Magnifications: 50 µm). **A** Normal group **B** Diabetic model **C** *B. subtilis* **D** ZBiotics 0.5 ml/ kg **E** ZBiotics 1 ml/ kg. Intact tubular epithelium (arrow), intact renal corpuscles (red star), Vacuolar degenerative changes and nuclear pyknosis (red arrow), significant tubular dilation (star), inflammatory cells infiltrates (arrow head)

Normal control samples showed almost intact well organized morphological features of renal parenchyma. However, model samples revealed marked tubular degenerative changes records including most of cortical zone segments with vacuolar degenerative changes and nuclear pyknosis and significant tubular dilation. Moderate interstitial mononuclear inflammatory cells infiltrates were showed. *B. subtilis* treated samples almost showed the same histopathological changes. With ZBiotics, the dosage 0.5 ml treated samples showed apparent intact tubular epithelium in most of nephron segments with single records of tubular degenerative changes and mild luminal exfoliation of some epithelial cells. Moreover; minimal inflammatory infiltrates were observed. ZBiotics 1 ml treated samples showed more organized histological features yet with persistent mild luminal exfoliation of some epithelial cells and minimal inflammatory cells infiltrates (Fig. 4).

**Fig. 5:**
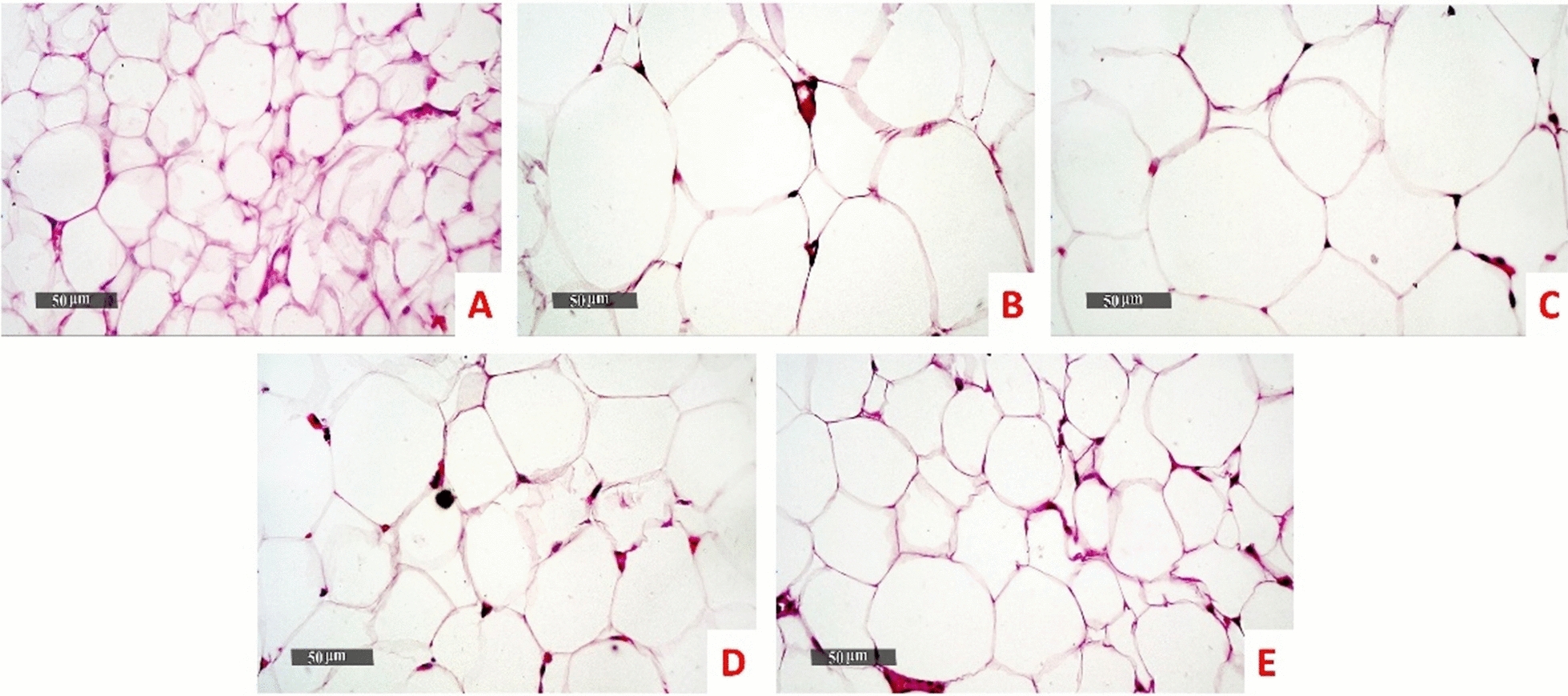
The effect of diabetic settings and ZBiotics use on adipose tissues using HE (Magnifications: 50 µm). **A** Normal group **B** Diabetic model **C** *B. subtilis* **D** ZBiotics 0.5 ml/ kg **E** ZBiotics 1 ml/ kg

**Fig. 6:**
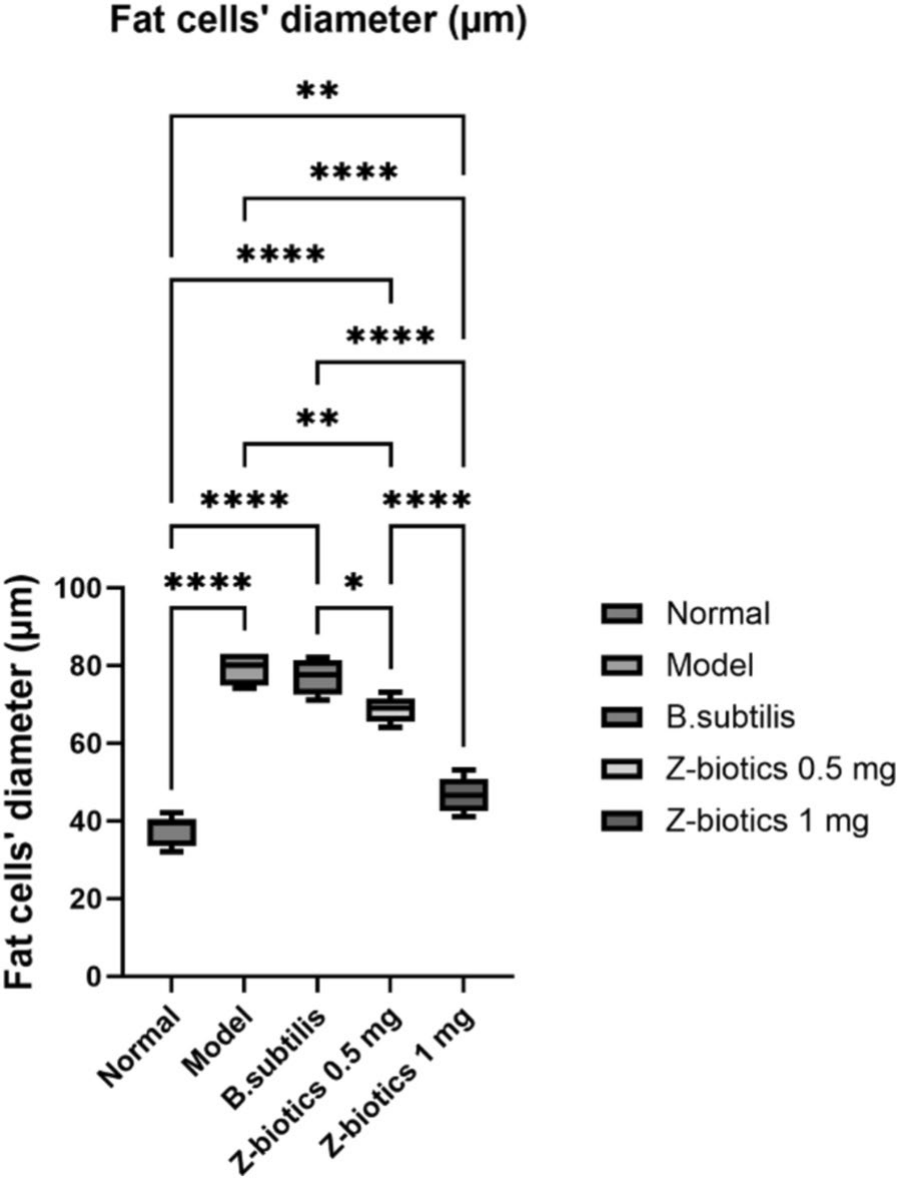
Morphometric analysis of adipocytes: both ZBiotics treated groups showed significant decline in adipocyte sizes compared with the model samples (****p < 0.0001)

Normal control samples showed almost intact well organized morphological features of white adipocytes giving a normal unilocular appearance. In-between loci, blood vessels and minimal collagen fibers were distributed. In contrast, model samples showed blood vessels and collagen fibers were squeezed out leaving large empty loci of white adipocytes. With morphometric analysis of adipocyte diameter, their sizes were significantly two folds larger than that of normal samples (p < 0.0001). In comparison to model samples, the same picture was detected with *B. subtilis* samples. Z-Biotic 0.5 ml showed significant decline in adipocyte sizes (p < 0.001) and so did ZBiotics 1 ml dosage (p < 0.0001) compared with the model samples. Moreover, Samples treated with ZBiotics 1 ml microscopically showed regaining of the inter-adipocytic blood vessels and collagen fibers (Figs. 5, 6).

### Immunohistochemical investigations of LC3 and TNF-a

**Fig. 7:**
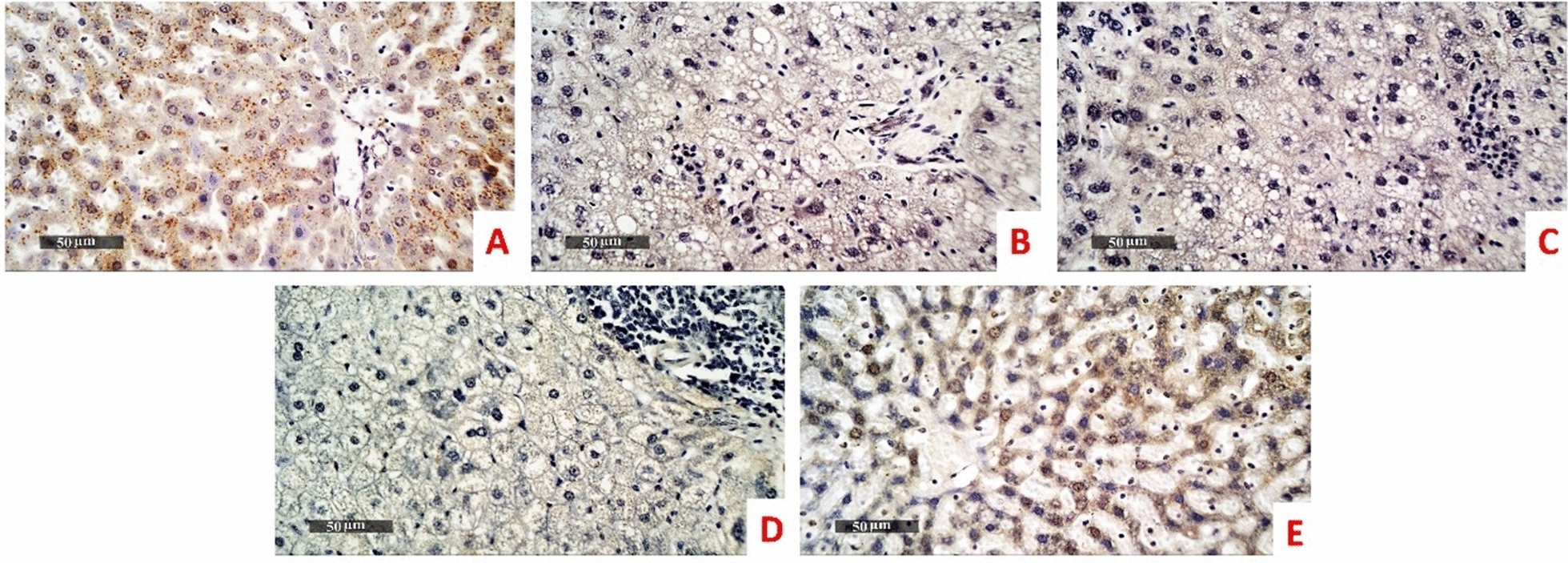
Immunohistochemical area percentage-based quantitative analysis of LC3B in liver tissues (Magnifications: 50 µm). **A** Normal group **B** Diabetic model **C** *B. subtilis* **D** ZBiotics 0.5 ml/ kg **E** ZBiotics 1 ml/ kg

**Fig. 8:**
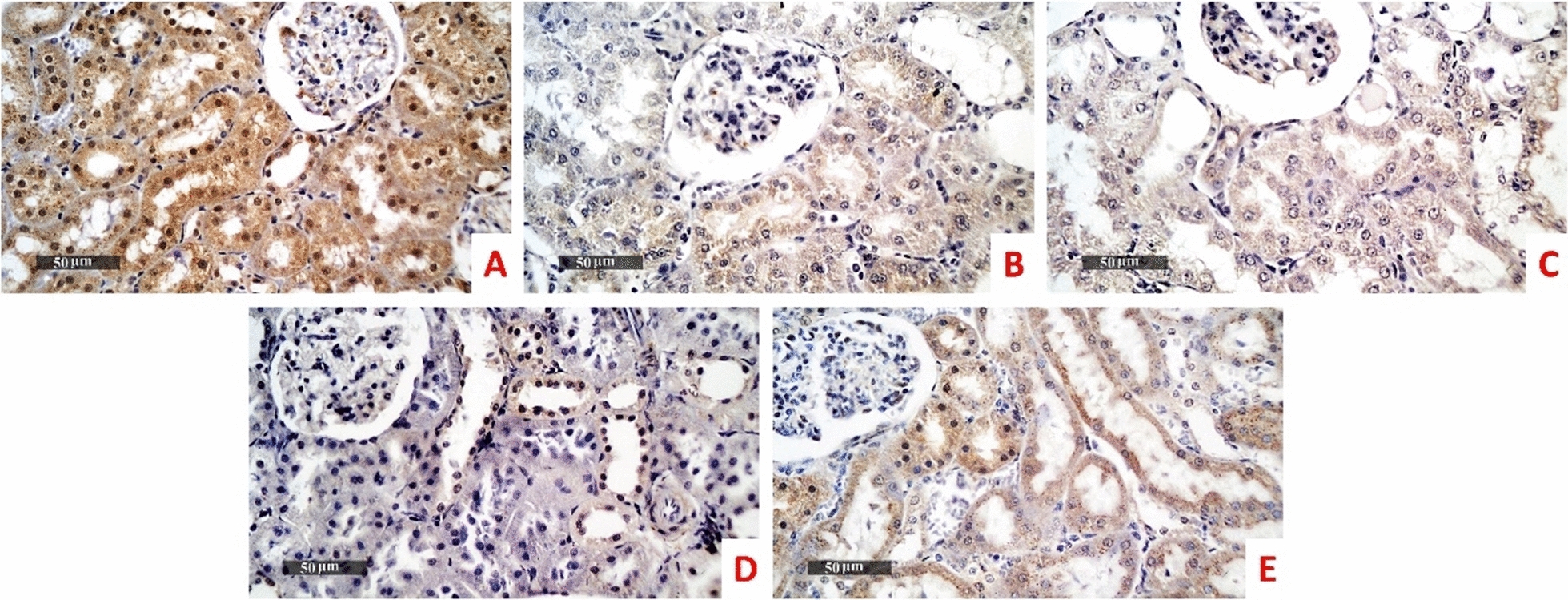
Immunohistochemical area percentage-based quantitative analysis of LC3B in kidney tissues (Magnifications: 50 µm). **A** Normal group **B** Diabetic model **C** *B. subtilis* **D** ZBiotics 0.5 ml/ kg **E** ZBiotics 1 ml/kg

**Fig. 9:**
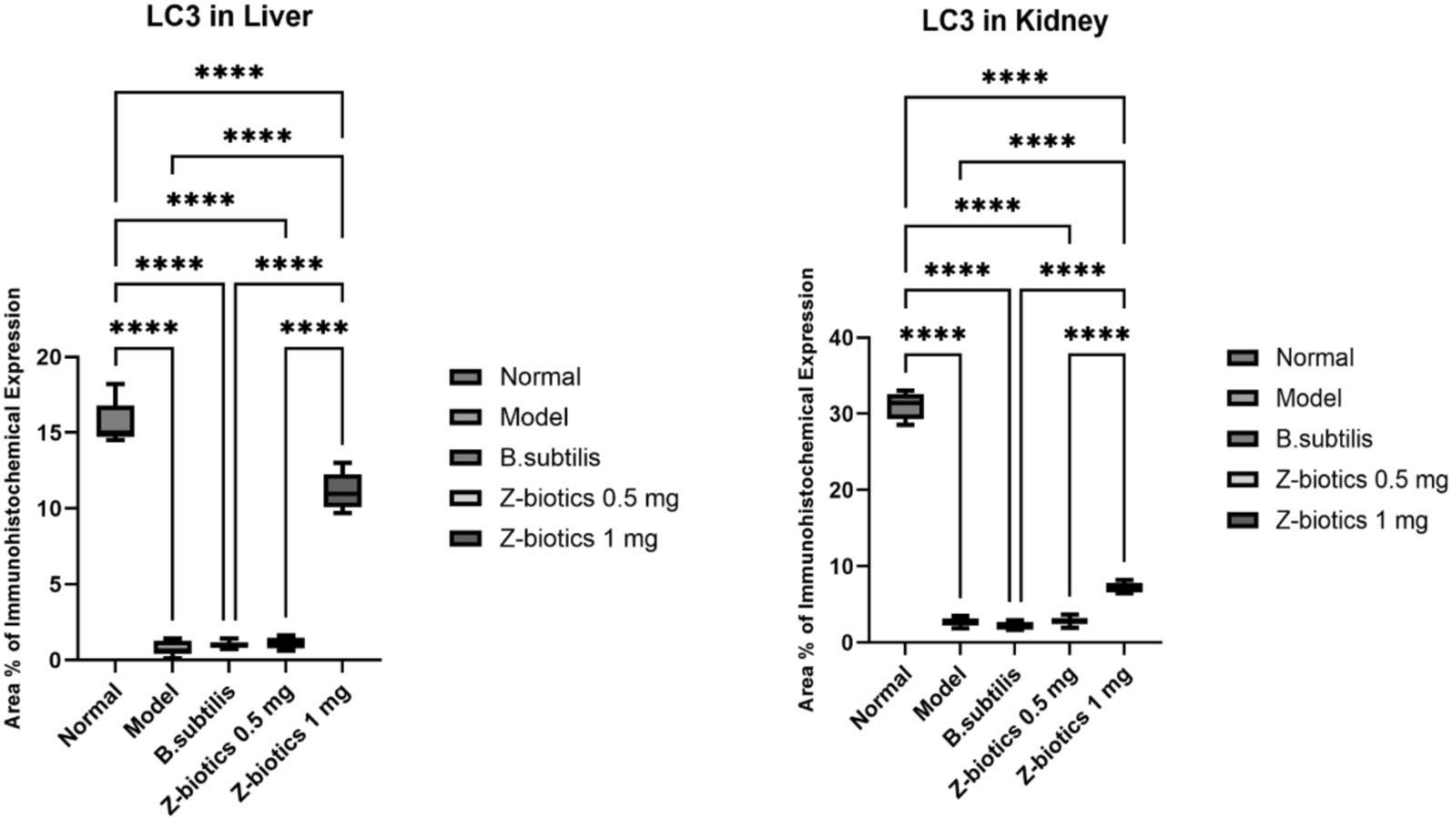
LC3 expression levels in liver tissues and kidney tissues: ZBiotics with the dosage 1 ml/ kg elevated autophagy LC3 marker to a significant level when compared to that of the diabetic models (****p < 0.0001)

Immunohistochemical area percentage-based quantitative analysis revealed highly significant decrease of LC3 expression level in the model group (mean area percentage = 0.733% and 2.617% in liver and kidney tissues respectively) compared with normal control (15.63% and 31.05% in liver and kidney tissues respectively). The LC3 expression level in *B. subtilis* group didn’t significantly differ in comparison to the model the group (mean area percentage = 1.000% and 2.05% in liver and kidney tissues respectively). Administration of ZBiotics 1 ml/ kg could significantly rise back up the LC3 expression levels in liver (Figs. 7, 9) and kidney tissues (Figs. 8, 9) (p < 0.0001). However, ZBiotics 0.5 ml/ kg failed to significantly augment the LC3 expression in rats’ liver tissues; mean area percentage = 0.733% in the model group and mean area percentage = 1.083% in the liver tissues treated with ZBiotics 0.5 ml/ kg (p value = 0.96 with Tukey’s multiple comparisons test). Likewise, ZBiotics 0.5 ml/ kg couldn’t significantly elevate the LC3 expression level in the treated kidney tissues (mean area percentage = 2.817%) compared with the model group kidney tissues (mean area percentage = 2.617%). Both in liver and kidney tissues, the ZBiotics dosage 1 ml/ kg showed a highly significant advantage over the ZBiotics dosage 0.5 ml/ kg as regards the elevation of LC3 expression level (p value < 0.0001).

**Fig. 10:**
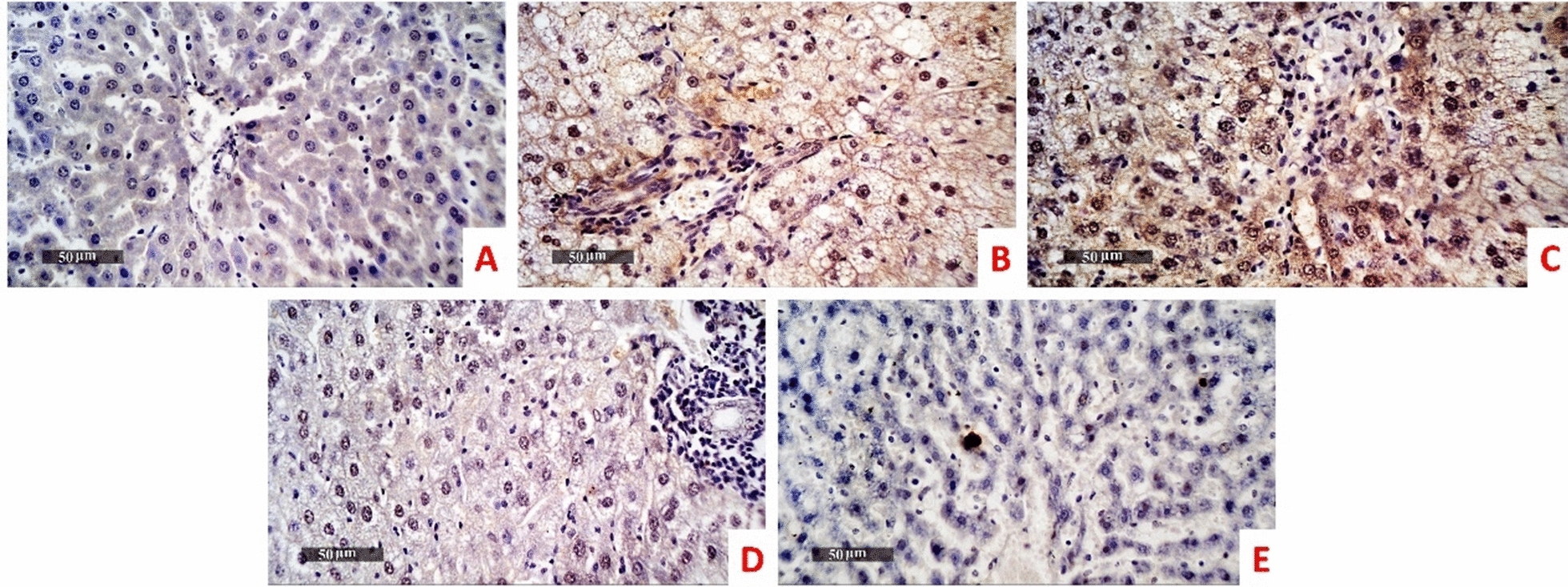
Immunohistochemical area percentage-based quantitative analysis of TNF-a in liver tissues (Magnifications: 50 µm). **A** Normal group **B** Diabetic model **C** *B. subtilis* **D** ZBiotics 0.5 ml/ kg **E** ZBiotics 1 ml/ kg

**Fig. 11:**
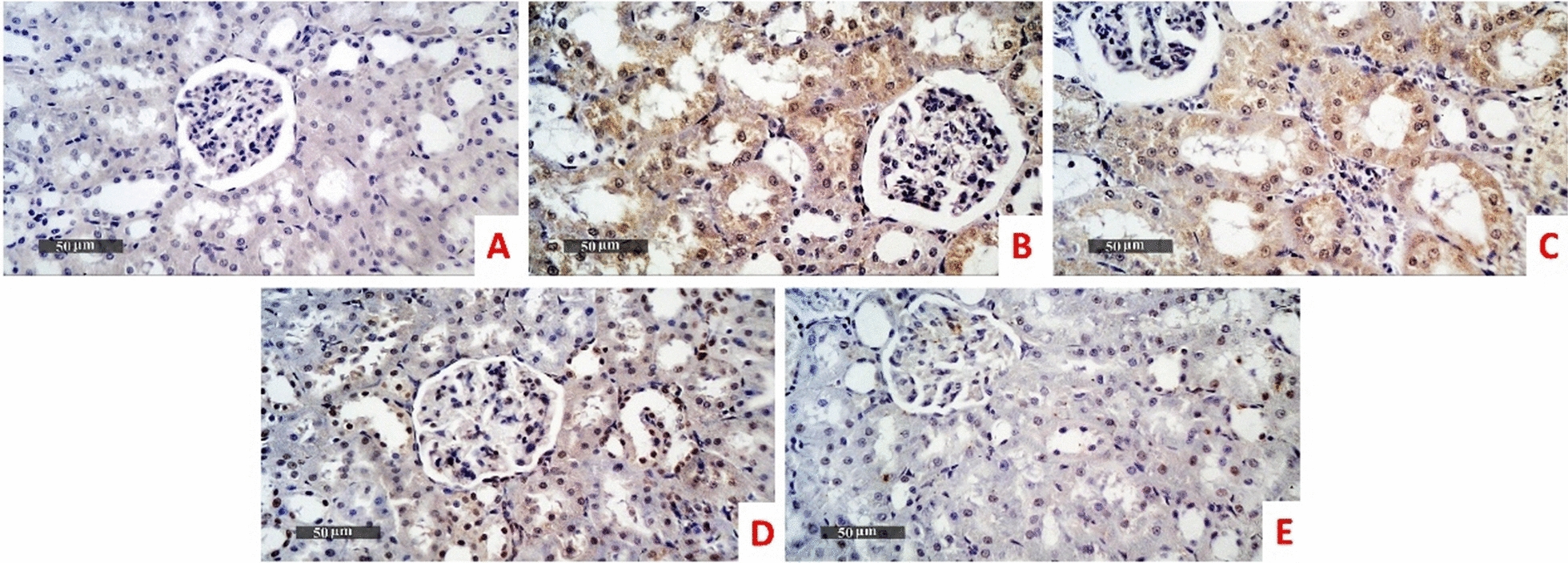
Immunohistochemical area percentage-based quantitative analysis of TNF-a in kidney tissues (Magnifications: 50 µm). **A** Normal group **B** Diabetic model **C** *B. subtilis* **D** ZBiotics 0.5 ml/ kg **E** ZBiotics 1 ml/ kg

**Fig. 12:**
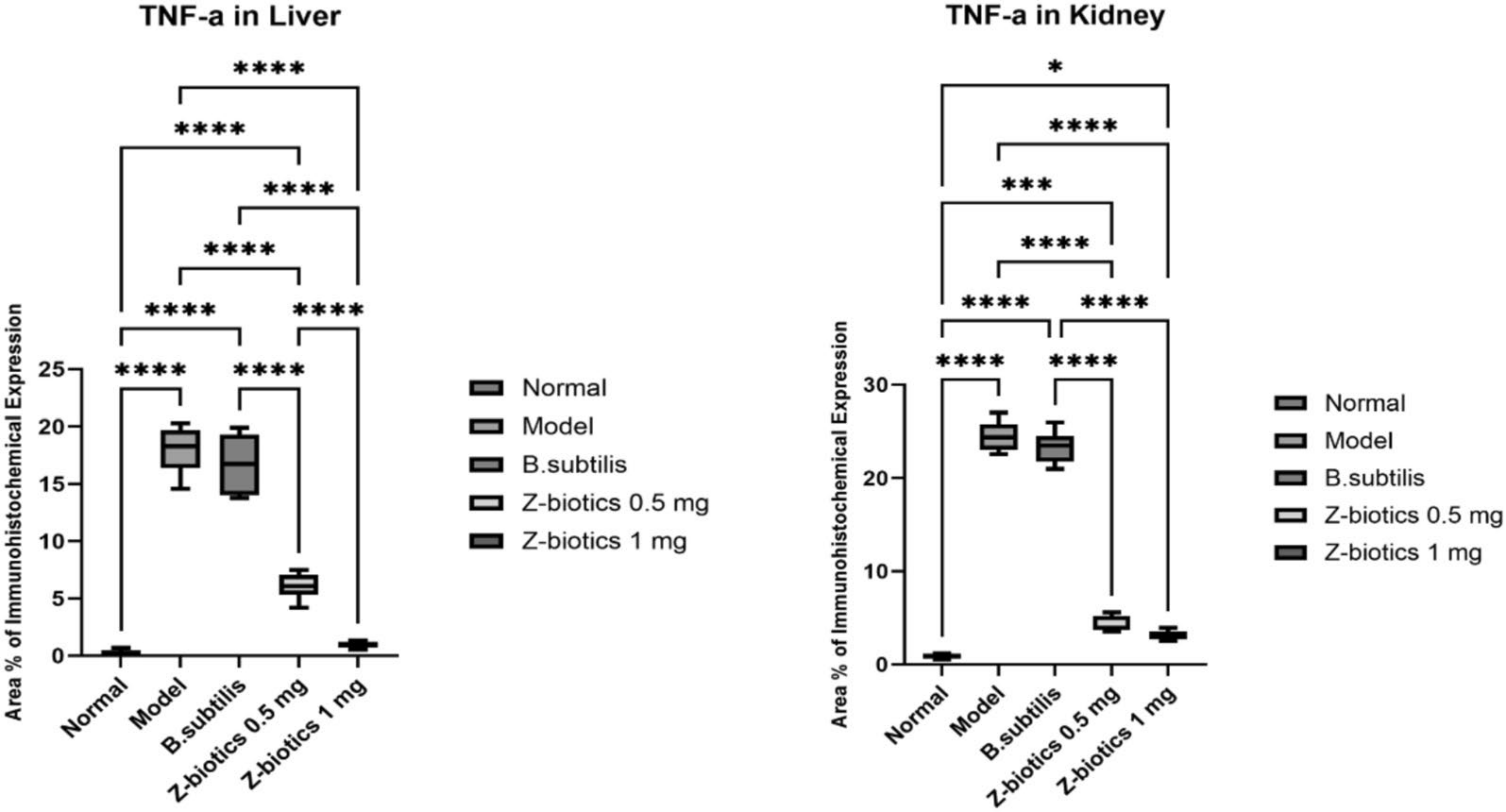
TNF-a expression levels in liver tissues and kidney tissues: both ZBiotics treated groups showed TNF-a level suppression to a significant level when compared to that of the diabetic models (****p < 0.0001)

For the expression level of TNF-a, immunohistochemical area percentage-based quantitative analysis revealed highly significant upsurge of TNF-a expression level in the model group (mean area percentage = 18% and 24.5% in liver (Fig. 10) and kidney tissues (Fig. 11) respectively) compared with normal control (0.283% and 0.883% in liver and kidney tissues respectively). The TNF-a expression level in *B. subtilis* group didn’t significantly differ in comparison to the model the group (mean area percentage = 16.7% and 23.3% in liver and kidney tissues respectively). Administration of ZBiotics 1 ml/ kg could significantly suppress the TNF-a expression levels in liver and kidney tissues (p < 0.0001). ZBiotics 0.5 ml/ kg, when compared with the model group, carried the same effect with a similar statistical significance (p < 0.0001). In liver tissues, the ZBiotics dosage 1 ml/ kg showed a highly significant advantage over the ZBiotics dosage 0.5 ml/ kg as regards the suppression of TNF-a expression level (p value < 0.0001). However, in kidney tissues, the mean difference of area percentage between the ZBiotics 0.5 ml/ kg treated group and the ZBiotics 1 ml/ kg treated group = 1.2% with p value = 0.35 (> 0.05) (Fig. 12).

### Effect of ZBiotics on biochemical parameters and rats’ body weights

**Fig. 13:**
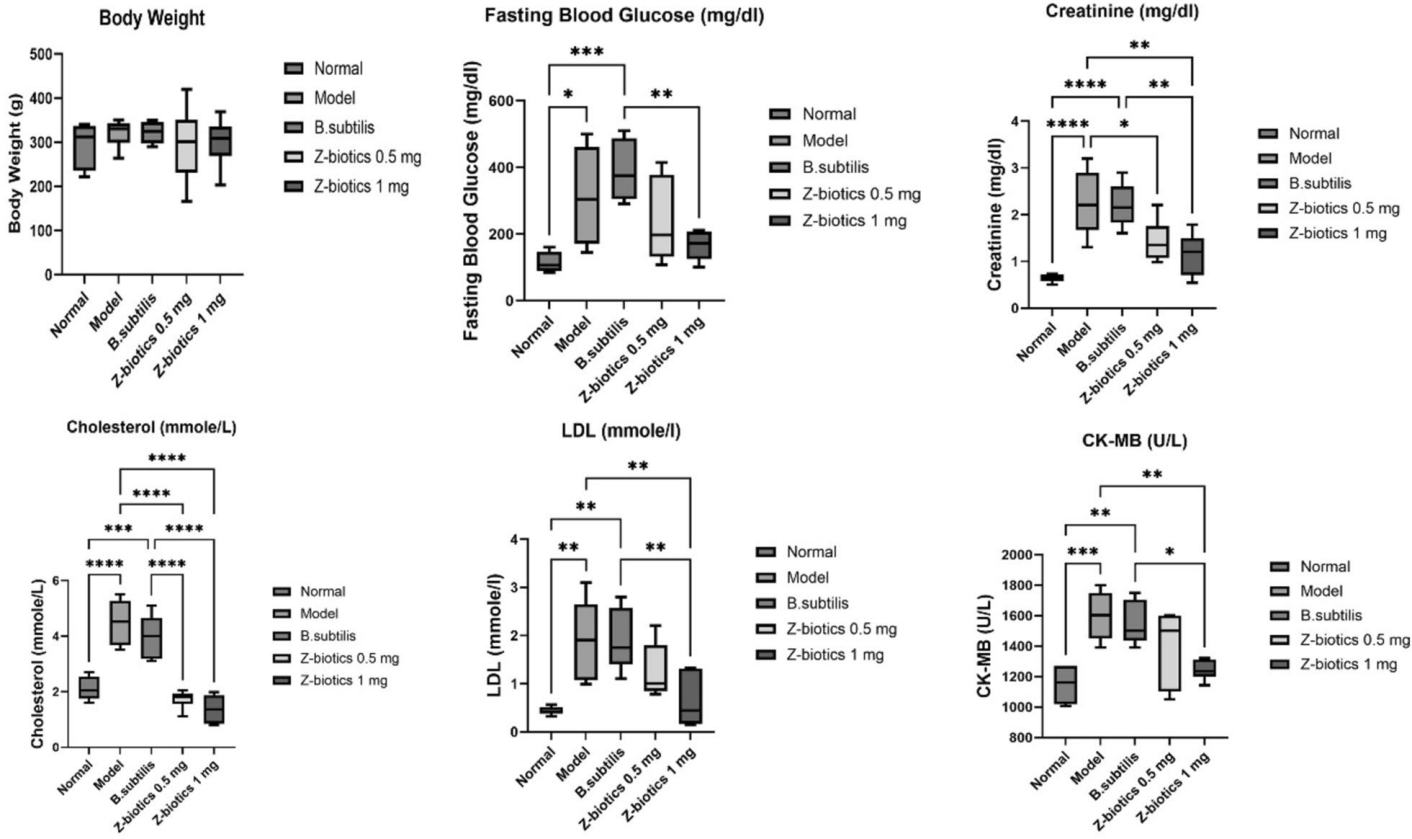
Showing effect of ZBiotics on rats’ body weights, fasting blood glucose, creatinine, cholesterol, LDL and CK-MB. Values are mean ± SD; number of animals = 6 rats/each group. ****p < 0.0001. ***p < 0.001, **p < 0.01, *p < 0.05. Stats performed by One-way ANOVA followed by Tukey’s multiple comparison test

STZ/HFD administration resulted in highly significant (p < 0.0001) augmentation in cholesterol, creatinine level and urinary albumin/ creatinine ratio. A very significant (p < 0.001) increase in CK-MB was noticed. Triglycerides, HDL, LDL, urea nitrogen in addition to fasting blood glucose were significantly elevated in the diabetic model group when compared with the normal group. Moreover, diabetic rats, when treated with ZBiotics 0.5 ml/ kg, caused a considerable decrease only in cholesterol (p < 0.0001) and creatinine levels (p < 0.05). ZBiotics 1 ml/kg demonstrated an advantage over the 0.5 mg/kg dosage. It caused a highly significant (p < 0.0001) decline in HDL and significant decrease in LDL and CK-MB (p < 0.01), Triglycerides and Urea nitrogen (p < 0.05). Nor the ZBiotics dosage 0.5 or 1 mg/ kg could significantly lessen the fasting blood glucose level or reverse the urinary albumin/ creatinine ratio back to the normal group level. Also, with Tukey’s multiple comparisons test, no statistical significance was reported for the mean differences of troponin level or rats’ body weights among the groups of experiment. In all comparisons, no mean differences were reported between model and *B. subtilis* groups (Fig. 13, Table 1).

**Table 1:** Effect of ZBiotics on rats’ body weights and biochemical parameters

Parameter	Groups									
	Normal		Model		*B. subtilis*		Z-biotics 0.5 ml		Z-biotics 1 ml	
	Mean	± SD	Mean	± SD	Mean	± SD	Mean	± SD	Mean	± SD
Body weight (g)	294	50.73	321	31.22	322.5	25.64	295	83.92	301	54.83
Cholesterol (mmole/L)	2.12	0.43	4.50	0.78	3.98	0.77	1.73	0.33	1.37	0.52
TG (mmole/L)	3.45	1.03	7.18	1.06	5.85	0.63	5.13	0.97	4.78	2.25
HDL (mmole/L)	0.75	0.36	1.35	0.33	1.48	0.15	0.99	0.26	0.27	0.06
LDL (mmole/L)	0.46	0.08	1.92	0.83	1.90	0.64	1.26	0.56	0.64	0.56
Creatinine (mg/dl)	0.65	0.09	2.25	0.73	2.20	0.46	1.43	0.45	1.15	0.45
Fasting blood glucose (mg/dl)	114.83	30.32	313.50	143.16	390.00	93.17	237.00	126.83	165.50	44.07
Urea nitrogen (mg/dl)	137.33	6.50	238.33	80.16	235.00	37.82	170.33	23.68	153.67	23.93
CK-MB (U/L)	1148.33	116.45	1600.00	160.88	1546.67	141.66	1395.33	245.60	1244.00	65.02
Troponin (ng/ml)	0.02	0.01	0.09	0.03	0.09	0.03	0.15	0.23	0.05	0.03
Urine albumin/creatinine ratio (mg/g)	9.17	0.94	52.80	19.33	54.60	12.68	44.78	8.78	45.35	9.14

Values are mean ± SD; number of animals = 6 rats/each group. Stats performed by one-way ANOVA followed by Tukey’s multiple comparison test

### Effect of ZBiotics on molecular parameters (lnc-RP4-605O3.4, miR_611, NFKBI and CHUK)

**Fig. 14:**
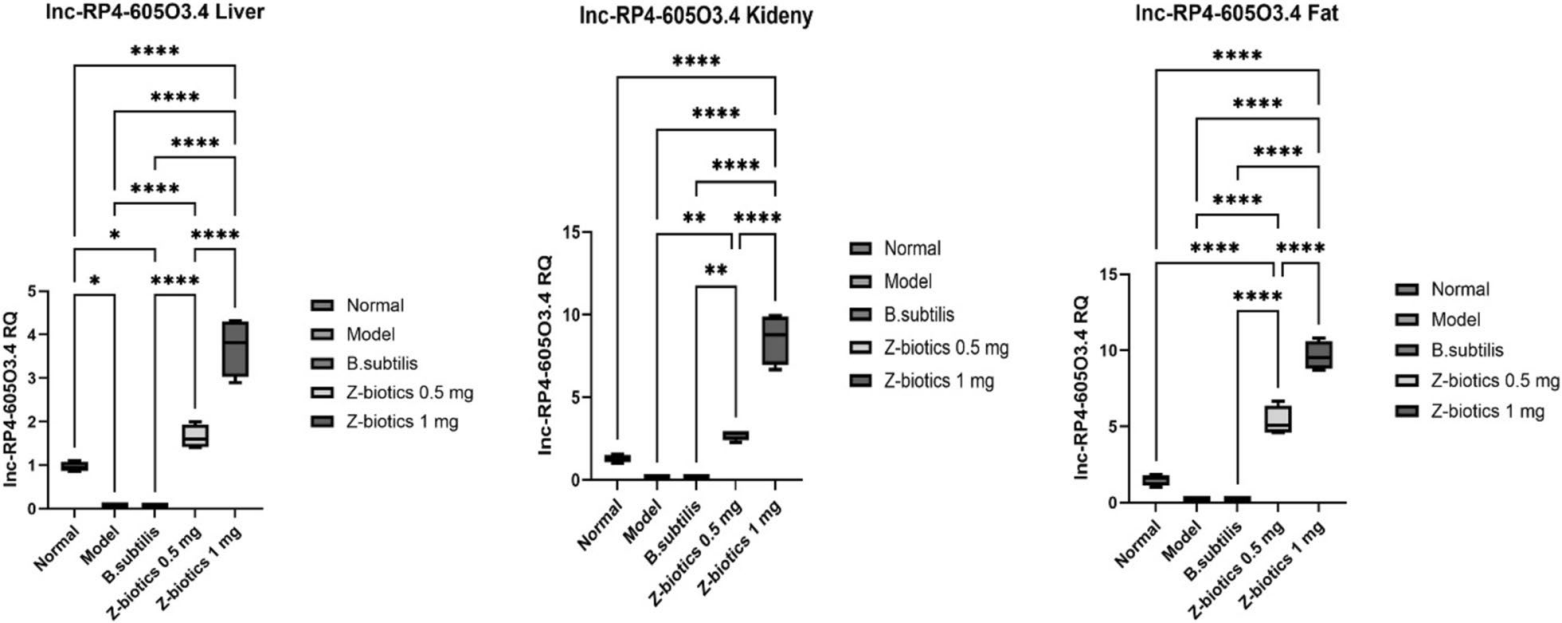
Showing effect of ZBiotics on lnc-RP4-605O3.4 expression levels in liver, kidney and adipose tissues. Values are mean ± SD; number of animals = 6 rats/each group. ****p < 0.0001. ***p < 0.001, **p < 0.01, *p < 0.05. Stats performed by One-way ANOVA followed by Tukey’s multiple comparison test

For lnc-RP4-605O3.4, in kidney and fat tissues, the model group proved no statistical significance as regards the difference in the means of the gene relative quantification (RQ) when compared with the normal group. However, in liver tissues, STZ/HFD administration resulted in weak significant decline in the gene RQ when compared with the normal group (p value = 0.026). In comparison with the model group, the ZBiotics dosage 0.5 ml/kg significantly increased the gene RQ by 19.4 folds in liver tissues, 18.8 folds in kidney tissues and 24.4 folds in adipose tissues. Moreover, the ZBiotics dosage 1 ml/ kg significantly caused RQ upsurge, yet by 34 folds in liver tissues, 59 folds in kidney tissues and 44 folds in adipose tissues. In all tissues tested, the ZBiotics 1 ml/ kg showed highly statistically significant rise in the gene RQ over the dosage 0.5 ml/ kg (Fig. 14).

**Fig. 15:**
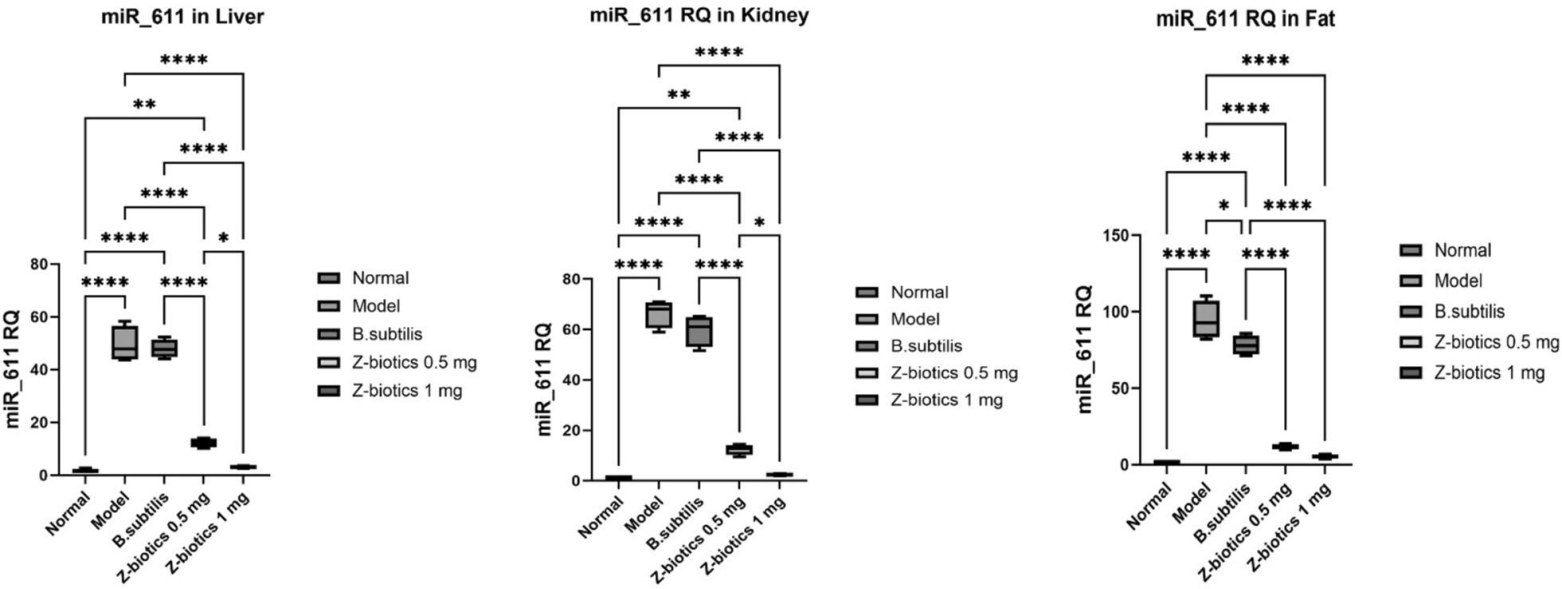
Showing effect of ZBiotics on miR_611 expression levels in liver, kidney and adipose tissues. Values are mean ± SD; number of animals = 6 rats/each group. ****p < 0.0001. ***p < 0.001, **p < 0.01, *p < 0.05. Stats performed by One-way ANOVA followed by Tukey’s multiple comparison test

For miR_611, in liver, kidney and fat tissues, when compared with the normal group, the model group significantly increased the gene RQ by 31 folds in liver tissues, 68.5 folds in kidney tissues and 107 folds in adipose tissues (p value < 0.0001). In liver and kidney tissues, no significant mean differences could be reported in the gene RQ between model and *B. subtilis* groups. However, in adipose tissues, *B. subtilis* group showed downregulation in the gene RQ by 82% when compared with model group (p value < 0.05). In comparison with the model group, the ZBiotics dosage 0.5 ml/kg significantly downregulated the gene RQ in all tissues tested (p < 0.0001). Moreover, the ZBiotics dosage 1 mg/ kg significantly caused RQ downregulation to much less levels than caused by the dosage 0.5 ml/ kg in liver and kidney tissues, yet in adipose tissues, no statistical significance could be proven for the mean differences in the gene RQ between the two groups (p value = 0.6) (Fig. 15).

**Fig. 16:**
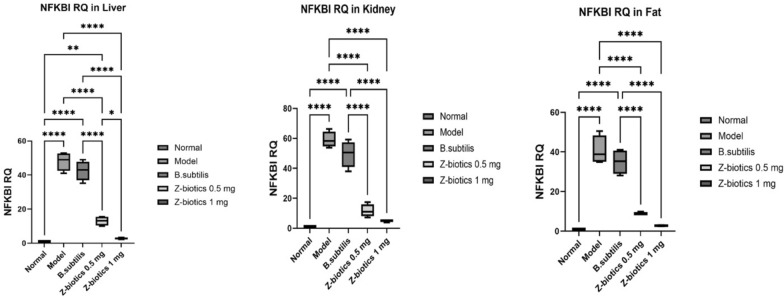
Showing effect of ZBiotics on NFKBI expression levels in liver, kidney and adipose tissues. Values are mean ± SD; number of animals = 6 rats/each group. ****p < 0.0001, ***p < 0.001, **p < 0.01, *p < 0.05. Stats performed by One-way ANOVA followed by Tukey’s multiple comparison test

For NFKBI, in liver, kidney and fat tissues, when compared with the normal group, the model group significantly increased the gene RQ by 50 folds in liver tissues, 51 folds in kidney tissues and 40.7 folds in adipose tissues (p value < 0.0001). No significant mean differences could be reported in the gene RQ between model and *B. subtilis* groups. In comparison with the model group, the ZBiotics dosage 0.5 mg/kg significantly downregulated the gene RQ in all tissues tested (p < 0.0001). Moreover, the ZBiotics dosage 1 ml/ kg significantly caused RQ downregulation to much less levels than caused by the dosage 0.5 ml/ kg in only liver tissues (p value < 0.01), yet in kidney and adipose tissues, no statistical significance (p values = 0.36 and 0.32 respectively) could be reported for the mean differences in the gene RQ between the two groups (Fig. 16).

**Fig. 17:**
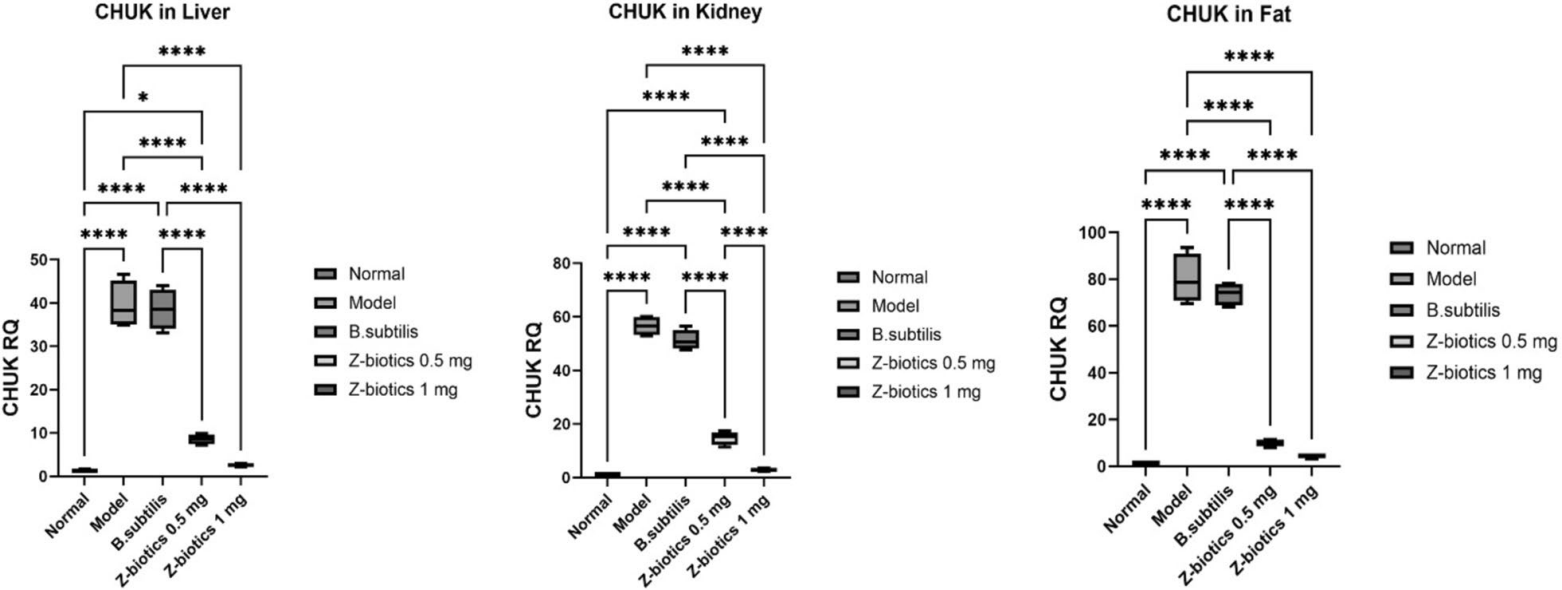
Showing effect of ZBiotics on CHUK expression levels in liver, kidney and adipose tissues. Values are mean ± SD; number of animals = 6 rats/each group. ****p < 0.0001, ***p < 0.001, **p < 0.01, *p < 0.05. Stats performed by One-way ANOVA followed by Tukey’s multiple comparison test

For CHUK, in liver, kidney and fat tissues, when compared with the normal group, the model group significantly increased the gene RQ by 31 folds in liver tissues, 58 folds in kidney tissues and 83.4 folds in adipose tissues (p value < 0.0001). No significant mean differences could be reported in the gene RQ between model and *B. subtilis* groups. In comparison with the model group, the ZBiotics dosage 0.5 ml/kg significantly downregulated the gene RQ in all tissues tested (p < 0.0001). Moreover, the ZBiotics dosage 1 ml/ kg significantly caused RQ downregulation to much less levels than caused by the dosage 0.5 ml/ kg in kidney tissues (p value < 0.0001), yet in liver and adipose tissues, no statistical significance (p values = 0.12 and 0.58 respectively) could be reported for the mean differences in the gene RQ between the two groups (Fig. 17).

## Discussion

Diabetes Mellitus type 2 is admittedly deemed one of the most prevalent non-communicable pandemics. It leads to dreadful systematic complications that could be precluded through combinatorial therapeutic approaches or at least retarded [22]. In the last decade, probiotics, themselves or by genetic engineering to acquire certain characteristics, have gained a reputation, grounded in time, to be effective in diabetes preclusive measures [26]. The present study showcased the crucial impact of one of the genetically engineered probiotics; ZBiotics^®^; which has been basicly manufactured for alcoholics to prevent hangover symptoms by the administration of acetaldehyde dehydrogenase enzyme (AcoD) gene in *B. subtilis* strains [18].

Acetaldehyde dehydrogenase enzyme (AcoD) gene in *B. subtilis* was driven from C. necator strains and when aligned with the homosapien form it showed ≈ 45% similarity [26]. The homosapien family of acetaldehyde dehydrogenase (or Aldehyde Dehydrogenase, ALDH) includes three members; ALDH1A1, ALDH2 and ALDH3A1. ALDH family exert beneficial efforts in scavenging oxidative stress operators such as Malondialdehyde (globally utilized as a marker for oxidative stress), 2-Alkenals and 4-HNE (which has cysteine-rich receptors on NF-KB inducing its inflammatory activity) [20]. ALDH2 is the most relevant member to the present study; it resides in mitochondria to operate the same way other ALDH family do to contain the potential ROS that would potentially harm mitochondria [21]. Massive mitochondrial damage by ROS is linked to diabetes mellitus type 2 [7]. Moreover, Kawasaki et al., have reported a linkage between ALDH2 deficiency and NASH [21] and Wang et al., have reported a linkage with the left ventricular damage with prolonged hyperglycemic settings [30]. ALDH2 polymorphism was statistically reported with Type 2 diabetes mellitus among Japanese population and with a rise in insulin resistance (estimated by HOMA-IR levels) [22]. When *B. subtilis* strains were inoculated with AcoD gene, they resulted in a decline in ALT and AST enzymes reflecting a relief of liver tissues from the oxidative-stress-induced inflammation. Also, they caused a decline in the levels of malondialdehyde and inflammatory mediators, for instance TNF-alpha [31]. Another patent genetically engineered *B. subtilis* strains to produce butyrate in a large amount to make advantage of butyrate as a short chain fatty acid (SCFA) to slash down visceral fat accumulation and insulin resistance [32, 33]. Yet, this is not the case with ZBiotics since *B. subtilis* itself produces little amounts of butyrate.

*Bacillus subtilis* are gram positive, aerobic and fast-growing bacteria that have the ability to ferment soy beans [34]. As a result, essential bioactive compounds are produced such as nattokinase, biogenic amines and isoflavones which are efficient inhibitors against mTORC1 which are inhibitors to autophagy via AKT pathway [35]; a mechanism to be the culprit behind the development of diabetes mellitus type 2 with obesity [9, 36]. Other compounds were proven to decline ROS and the associated inflammatory mediators e.g. TNF-alpha and NF-KB [34]. In contrast, the present study showed no impact of *B. subtilis* when administrated alone to rats. This could be briefed as the *B. subtilis* impact is attributed to its ability to ferment soy beans and not to *S. subtilis* biological impact itself.

**Fig. 18:**
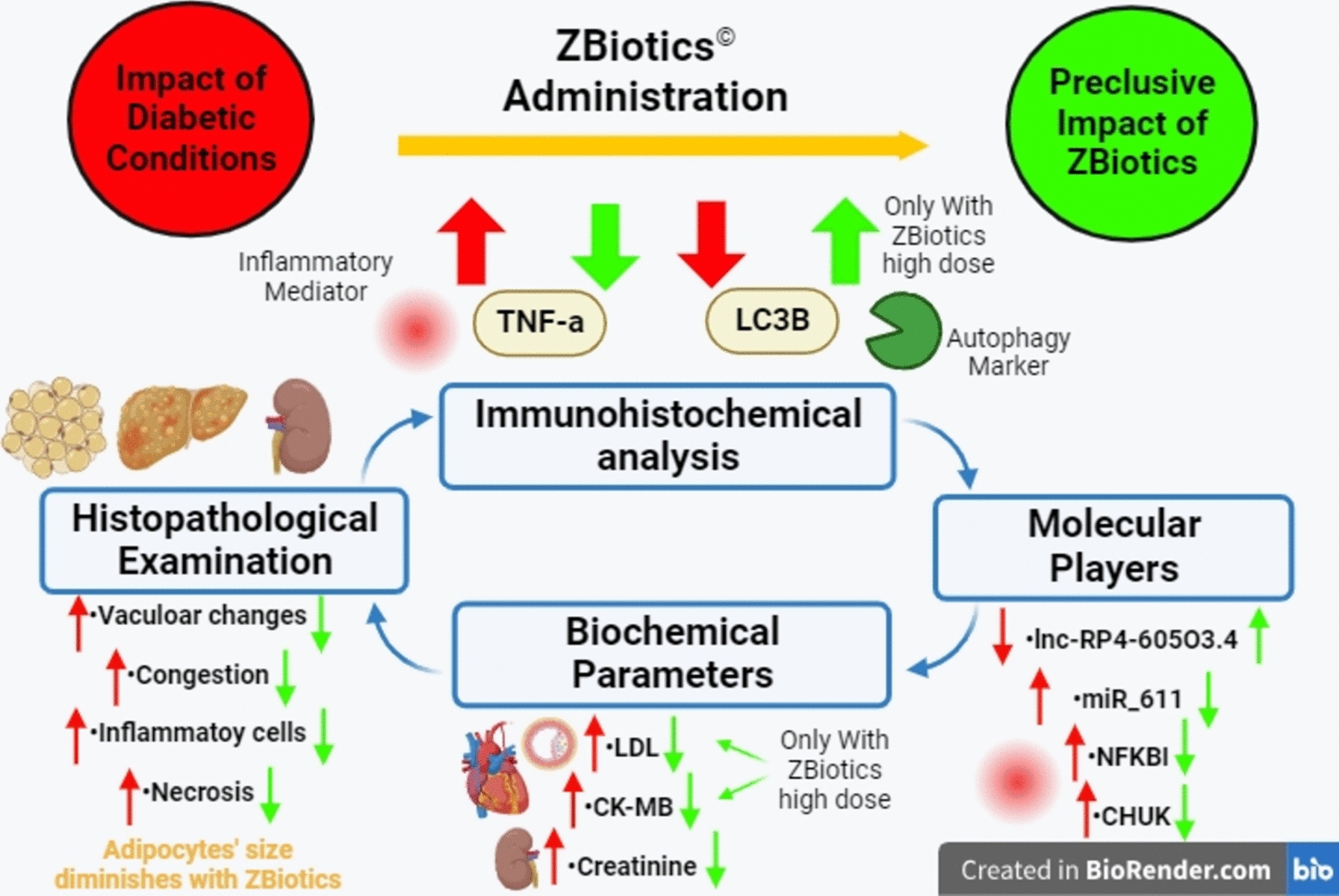
Concept map of the study hypothesis and results (Created by BioRender.com)

The impact of indigenous *B. subtilis* and acetaldehyde dehydrogenase can eloquently explain the results of the present study regarding how ZBiotics^®^ reversed the toll of diabetes on the studied histopathological, biochemical and molecular levels. The concept was based on two major mechanisms that could elicit inflammation that leads over time to diabetic settings and evolves diabetic complications; firstly, the oxidative-stress-induced inflammation, and secondly the disturbance in diabetic gut microbiota and barriers that could lead to the activation and cGAS-STING-NOD pathway and subsequently inflammation. We created diabetes mellitus type 2 model in rats with high fat diet (HFD) and streptozotocin and studied three organs reported to be a part of diabetes pathogenesis and complications (Fig. 18).

In liver tissues, Diabetes mellitus induces hepatocytic damage by mounting ROS and, in a vicious circle, the damaged hepatocytes worsen the diabetic conditions as liver is a central organ in glucose metabolism [37]. Accordingly, diabetic models demonstrated cellular degenerative changes and marked steatosis. In consistent with our mechanism of interest: diabetes associated inflammation, there was inter-hepatocytic infiltration of inflammatory cells, and upregulation of TNF-alpha expression level with immunohistochemistry. In contrast, in ZBiotics^®^ treated samples, this could be reversed to the normal tissue status, where there was normal liver architecture and low inflammatory cells with low TNF-alpha expression levels. As regards kidney tissues, a chronic status of hyperglycemia could elicit cellular damage by making use of the polyol pathway; glucose is converted to sorbitol by aldose reductase using NADPH, sorbitol should be converted to fructose by sorbitol dehydrogenase which is deficient in kidney. This, in turn, leads to accumulation of sorbitol (damaging effect owing to osmotic stress) and consumption of NADPH (deficient glutathione peroxidase activity on oxidative stress operators). The synergetic deficit leads to degenerative cellular damage eventually [38] and this could be detected in the present study with kidney tissues of diabetic models. Moreover, inflammatory cell infiltrates and expression levels of TNF-alpha were in the rise, and the whole picture could be reversed with ZBiotics^®^. Immunohistochemical analysis of LC3 protein as a marker of autophagy showed an eclipse of its level in diabetic model including liver and kidney tissues. This go hand in hand with the literature; autophagy is deficient in diabetes mellitus, obesity and NASH [9]. Also, autophagy recovery is reported to be an essential target for diabetes mellitus therapeutics [39]. Contrarily, ZBiotics^®^, albeit with the dosage 1 ml/kg, could elevate autophagy LC3 marker to a significant level when compared to that of the diabetic models.

Regarding adipose tissues, morphometric analysis showed bulging of adipocytes with diabetic models owing to the intracellular lipid build-up. The interplay of this phenomenon with diabetes can be illustrated as follows: lipogenesis occurs due to an abundance of insulin and NADH with chronic hyperglycemia, and when an abundance of fat is deposited intracellularly, this could modulate a series of inflammatory operators to cause insulin resistance consequently. For instance, diacylglycerol (DAG) activates PKC family (e.g. PKC θ) and ceramide induces JNK [40]. Both enzyme families can inactivate IRS1 substrates through the phosphorylation of serine and threonine residues. Moreover, excessive lipogenesis triggers the release of IL-6 and CRP which maneuver the pancreatic B-cells degenerative processes and preclude the regenerative ones [41]. Lipotoxicity can aggravate the glucotoxicity toll on mitochondria by surging ROS with subsequent induction of inflammosomes and inactivation of IRS1 [42]. These mechanisms could illustrate the ensuing changes in the present study regarding the histopathological changes in liver and kidney tissues as well as TNF-alpha expression levels. Interestingly, the increase in adipocyte diameter could be significantly minimalized by ZBiotics^®^ treatment.

Considering the changes at the biochemical parameters, a handful of remarks are worth to be noted. Diabetic models led to an increase in cholesterol, triglycerides and LDL-c levels and they demonstrated an elevation in creatinine and urine albumin/ creatinine ratio. Diabetes impact on CK-MB was totally expected; the CK-MB rise could be attributed to the diabetes-induced coronary vessels’ changes. Besides, it’s worthwhile noting that ZBiotics^®^ in the dosage 1 mg/kg could abandon the rise in LDL-c and CK-MB levels. In the light of the studied mechanisms herein, this could be argued as follows: LDL-c is hardwired into an increase in liver malondialdehyde levels which are oxidative stress markers. Besides, LDL-c could stimulate the clonal conversion of monocytes to macrophages which produces an arsenal of cytokines involved in the systematic inflammation with diabetes [43]. One of them is TNF-alpha, the cytokine of interest in the present study. TNF-alpha not only modulates the migration and mitogenesis processes of vascular endothelial cells [5], but also helps the transcytosis of LDL-c into the subendothelial regions of diabetic blood vessels [44]. ZBiotics^®^ is speculated to decline the level of oxidative stress mediators by the action of acetaldehyde dehydrogenase, at one point, and by evacuating the adipocytes from lipids of all forms. The impact of LDL-c in the subendothelial regions leads us to the discussion of CK-MB level. CK-MB is recognized as a marker for myocardial injury, the thing that is enhanced by the deposition of LDL-c as plaques in coronaries. Moreover, oxidative stress operators can hasten the detrimental process. 4-HNE, one the operators, is linked to myocardial injury in mice with mutations in acetaldehyde dehydrogenase enzyme [45]. This provides an explanation why ZBiotics^®^ could preclude the CK-MB elevation level in the present study. In contrast, ZBiotics^®^ couldn’t slash down fasting blood glucose levels which can be interpreted as ZBiotics^®^ is able to narrow down the diabetes-induced complications rather than an absolute resolution of the hyperglycemic settings.

In the present study, we attempted to find out STING-NOD-IR RNA panel utilizing bioinformatic approaches. The reason why STING-NOD is chosen as a target is because the structure plays a crucial role in the mediation of inflammatory processes [46] either elicited by the microbial lipopolysaccharides (LPS) or the microbial DNA fragments [47]. A set of lnc-RNAs [48] as well as miRNAs [49] are linked to poor glycemic control by participating in systematic inflammation cascades [50]. Several literatures suggested the relation between susceptibility locus on rat chromosome 1 and diabetes mellitus and cholesterol metabolism [51, 52] Moreover, many of them are considered potential biomarkers for the diabetic conditions and early predictors for the diabetic complications [48]. By bioinformatics analyses, we hypothesized that lncRNA RP4-60503.4 and miR-611 are hardwired into CHUK and NF-KB1. In diabetic settings fostered by inflammation, lncRNA RP4-60503.4 and CHUK are suggested to be competitors by sharing similar miR-611 response elements. Therefore, when diabetic models showed a surge in CHUK RQ and a decline in lncRNA RP4-60503.4, this could be interpreted as CHUK is produced in a large amount by the action of STING as well as the role of lncRNA RP4-60503.4 which sequestered miR-611 that could degrade or suppress CHUK. The molecular changes could be reversed by ZBiotics^®^ treatment in a stepwise manner with increasing dosage. This solidifies the causal relationship between ZBiotics® and its impact on alleviating the inflammatory operation.

In spite of the potentially clinical benefits for ZBiotics, there are some limitations to be considered for future studies. Firstly, the study does not provide extensive clinical trials or large-scale data to substantiate the claims about ZBiotics^®^’s efficacy. Secondly, the findings might not be universally applicable, as the effects of probiotics can vary significantly depending on individual microbiomes. Also, potential long-term safety and adverse effects of ZBiotics^®^ as a therapeutic candidate are not discussed. Therefore, we recommend to conduct large-scale, randomized clinical trials to evaluate the efficacy and safety of ZBiotics^®^ in relieving inflammatory conditions and diabetes complications. And, to evaluate the use of ZBiotics^®^ in combination with conventional therapies for diabetes or other inflammatory diseases to determine the likely synergistic benefits.

## Conclusions

ZBiotics^®^ is the first engineered probiotic to be advertently utilized for relieving hangover symptoms. The present study encompasses grounded evidence that ZBiotics^®^ impact far stretches beyond only enabling alcoholics to tolerate more ounces of alcohol. The combinatorial action of its efficient components; *B. subtilis* microbial structure and acetaldehyde dehydrogenase could get rid of the inflammatory operators and narrow down the diabetes-induced dreadful complications. Therefore, ZBiotics^®^ is recommended for clinical trials as a separate candidate or as an adjuvant to the conventional therapy. Also, we suggest further engineering of ZBiotics^®^ with butyrate and isoflavones to potentiate its preclusive effect.

## Supplementary Information

Additional file 1. DOCX 3584 KB

Additional file 2. XLSX 4277 KB

Additional file 3. XLSX 7674 KB

## Data Availability

Data are available upon reasonable request. No datasets were generated or analysed during the current study.
