# Supplementary material for: ZBiotics ameliorates T2DM-induced histopathological damage in liver, kidney and adipose tissues by modulating the NOD-like receptor signaling in Wistar rats
Source: Diabetol Metab Syndr. 2025 Feb 4;17:45. doi: 10.1186/s13098-025-01600-3 (PMC11792315; doi:10.1186/s13098-025-01600-3)
Supplement: Supplementary file 1 — Additional file 1. DOCX 3584 KB [file 13098_2025_1600_MOESM1_ESM.docx]

Supplementary table 1: : Differentially expressed genes (DEGs) were identified using the GEO2Data base

| **Accession number** | **Platform** | **Organism** | **Experiment type** | **Design** | **Number of samples** | |
| --- | --- | --- | --- | --- | --- | --- |
|  | | | | | Case | Control |
| GSE78721 | GPL15207[PrimeView] Affymetrix Human Gene Expression Array | Homo sapiens | Expression profiling by array | 30 controls & 30 diabetic subjects undergoing femur bone surgery and 16 controls & 19 diabetic subjects undergoing abdominal surgery (two samples from each subject, one subcutaneous and one visceral fat biopsy were extracted). So total 130 samples were obtained and analyzed for genome –wide gene expression profile of Adipocytes and infiltration macrophages from three different depots of adipose tissue. | 68 | 62 |
| GSE104948 | GPL22945 [HG-U133_Plus_2] Affymetrix Human Genome U133 Plus 2.0 Array [CDF: Brainarray HGU133Plus2_Hs_ENTREZG_v19] | [Homo sapiens](https://www.ncbi.nlm.nih.gov/Taxonomy/Browser/wwwtax.cgi?mode=Info&id=9606) | Expression profiling by array | RNA from the glomerular compartment was extracted and processed for hybridization on Affymetrix microarrays, annotated using Human Entrez Gene ID custom CDF version 19. | 7 | 18 |

**
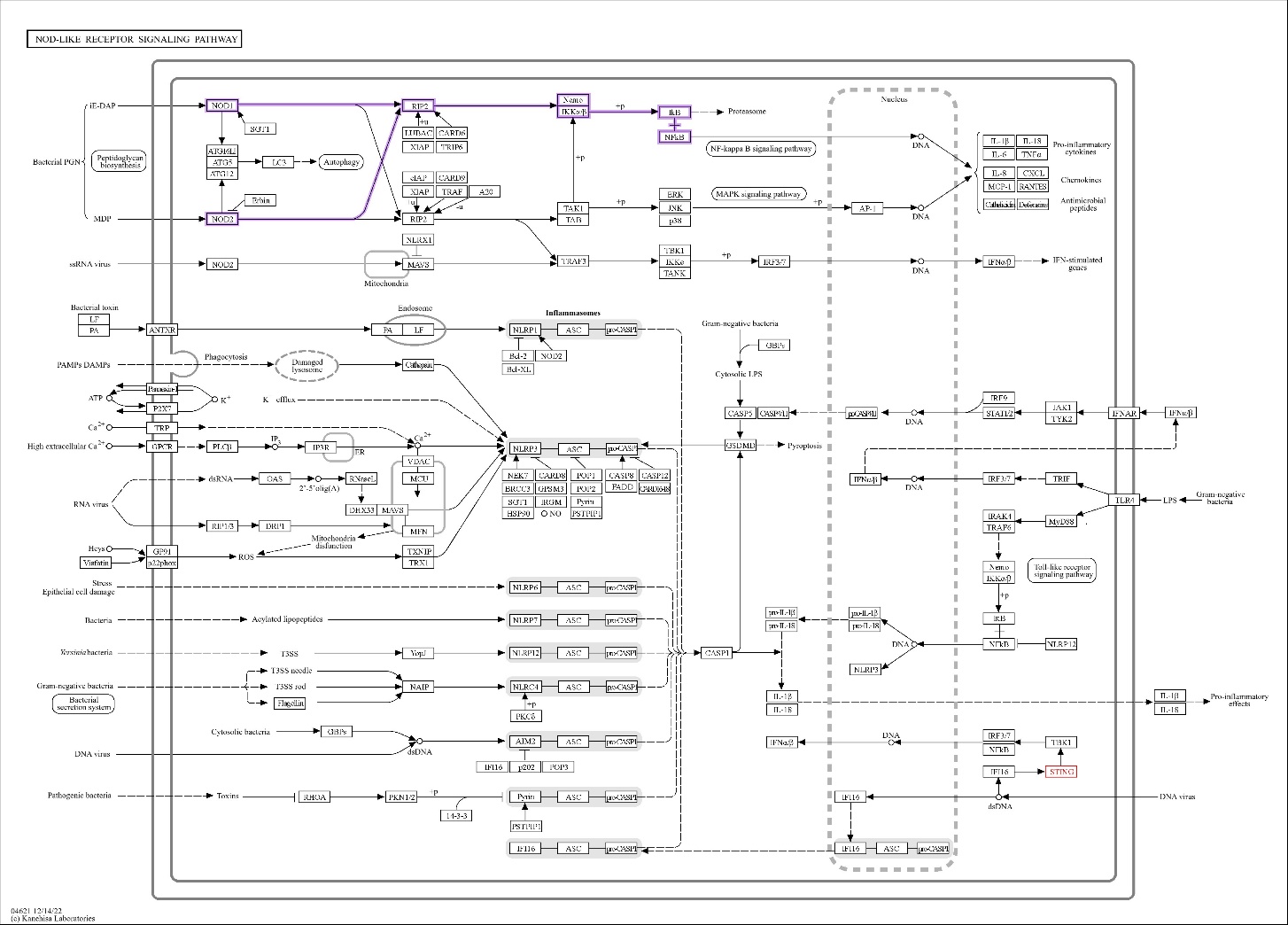
**

Fig. (S1) shows retrieval of a candidate gene: *CHUK* mRNAs related to insulin resistance relevant NLR signaling pathway from public microarray databases available at KEGG: Kyoto Encyclopedia of Genes and Genomes


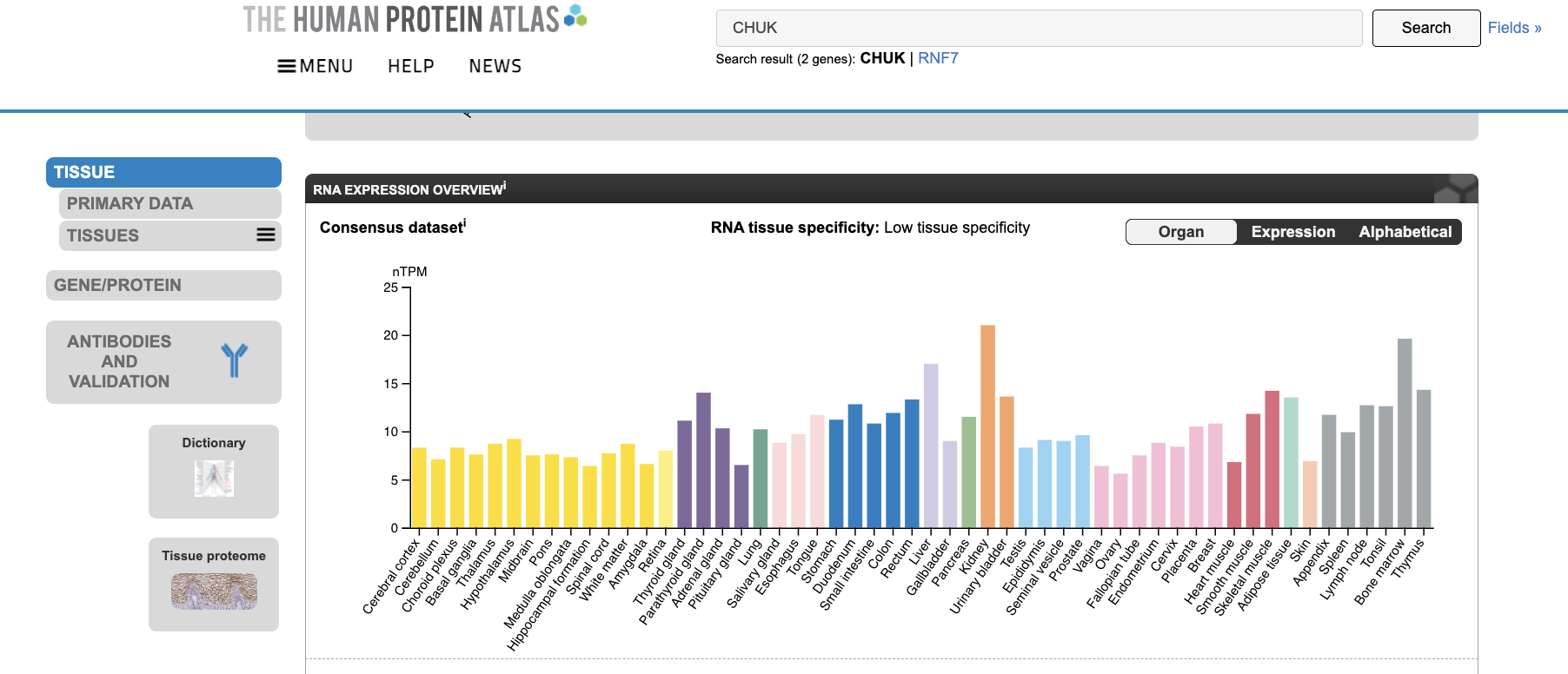


Fig. (S2) shows verification of the identified candidate gene (CHUK) expression in liver, kidney and adipose tissues through Human protein atlas


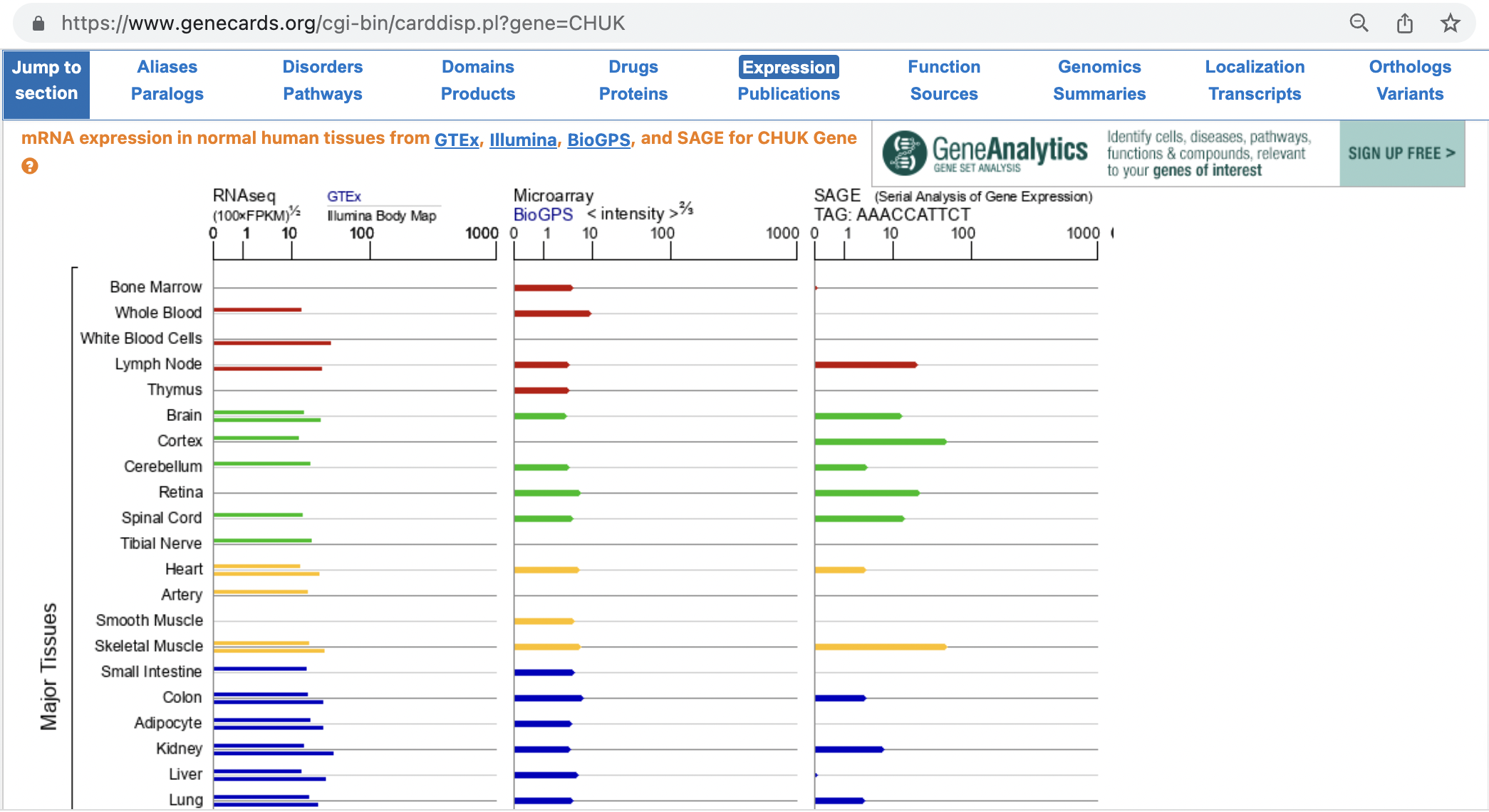


Fig. (S3) shows verification of the identified candidate gene (CHUK) expression in liver, kidney and adipose tissues via GeneCards Human Genes database


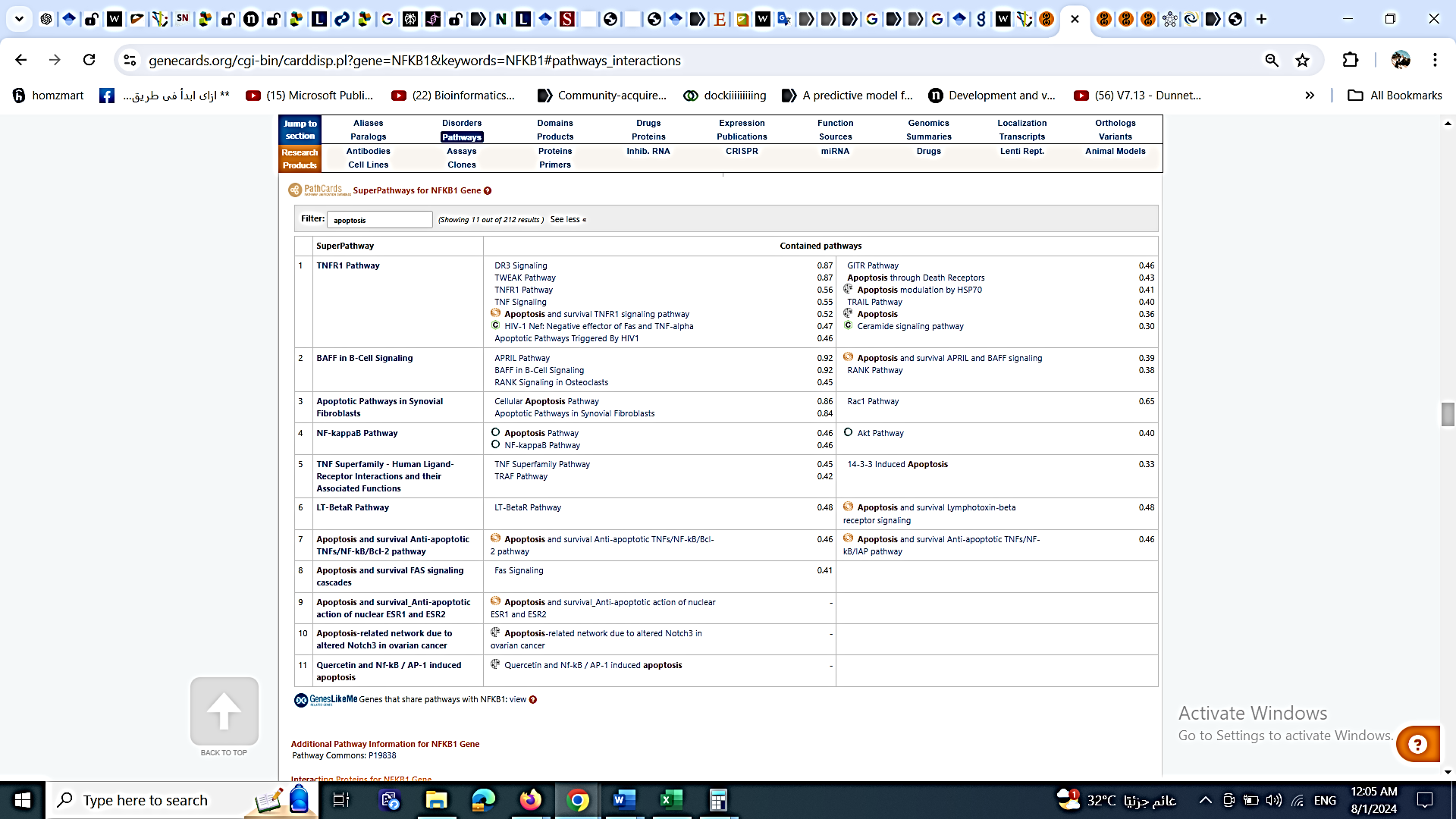


Fig. (S4) shows NFKB1 Gene Ontology using Genecards data base


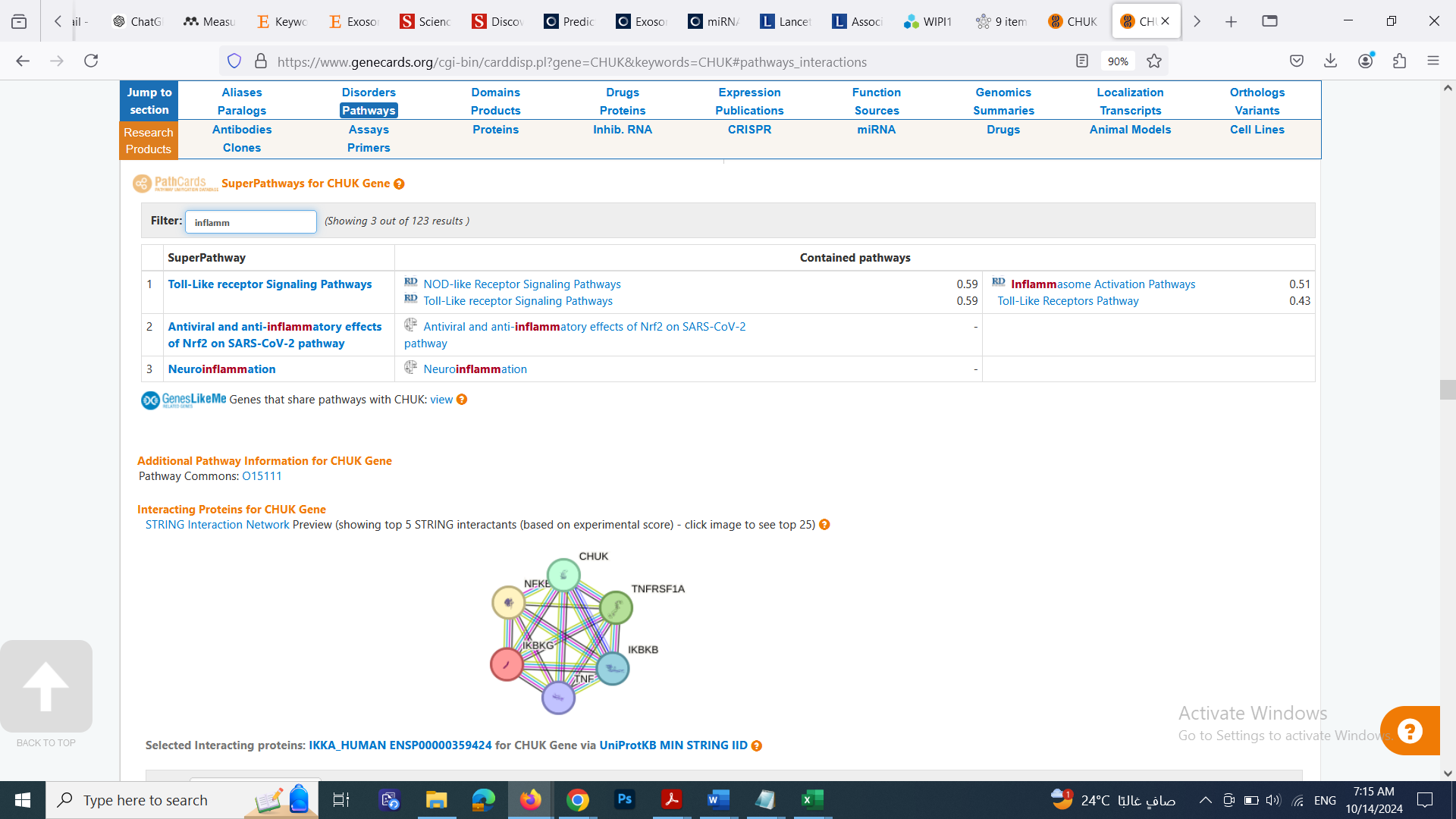


Fig. (S5) shows CHUCK Gene Ontology using Genecards data base


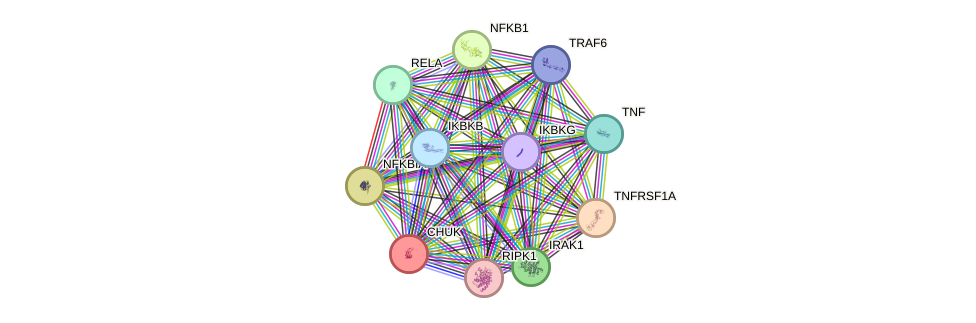


Fig. (S6A) shows the interaction between CHUK and NFKB1 in homosapiens through STRING database


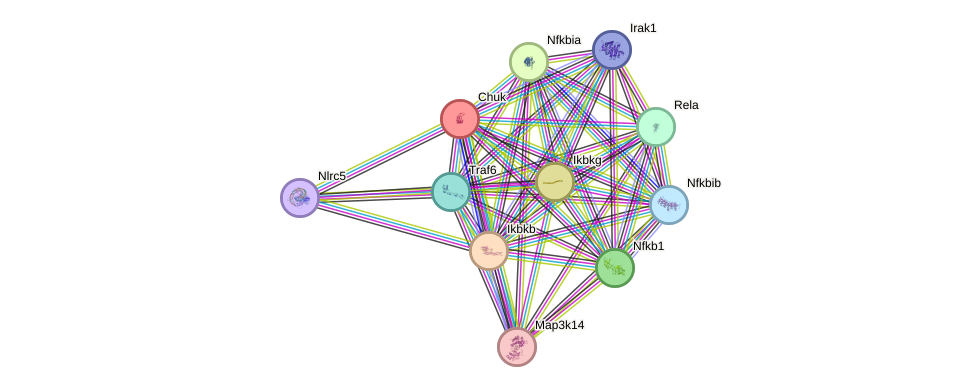


Fig. (S6B) shows similar interaction between CHUK and NFKB1 in rats through STRING database

*
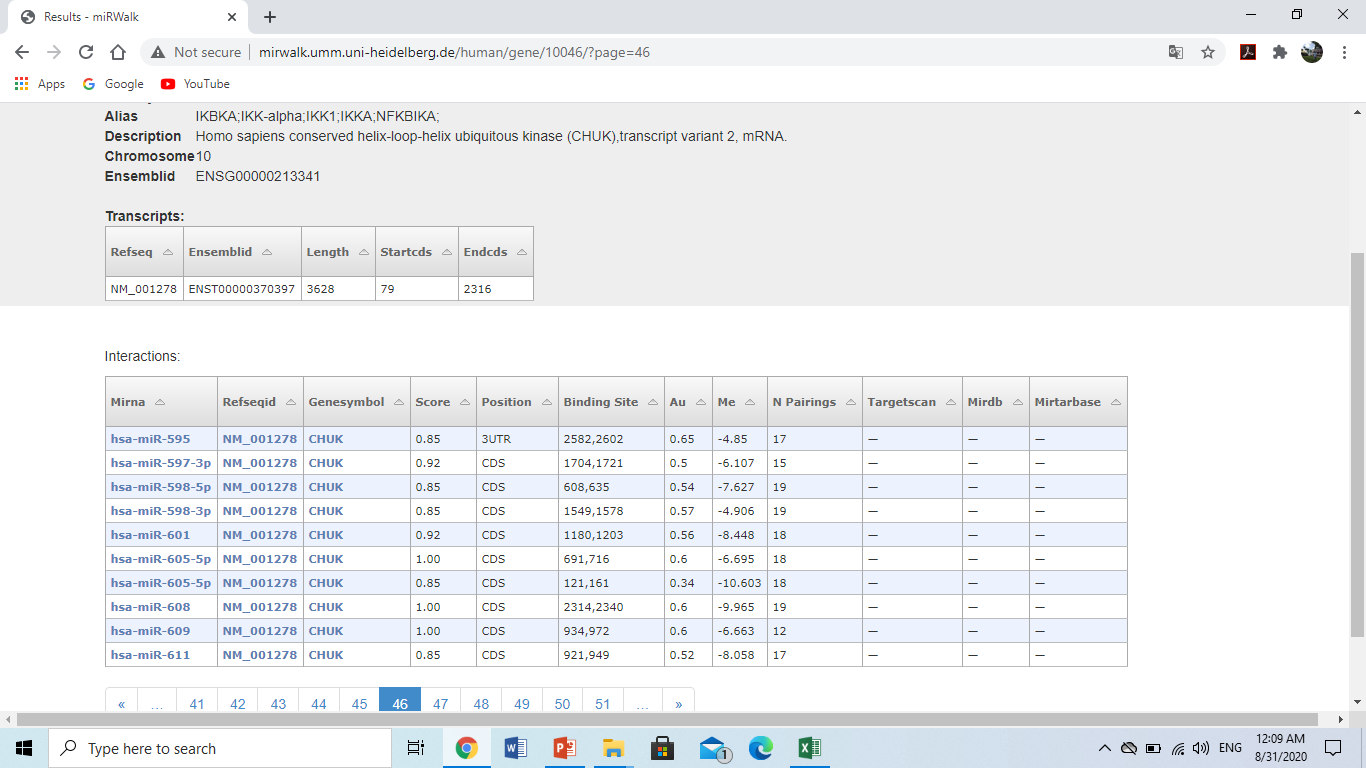
*

Fig. (S7) shows retrieval and Verification of hsa-miR-611 that act as epigenetic regulator for CHUK mRNA in IR at mirwalk database

**
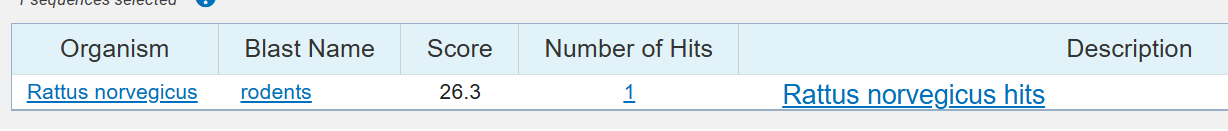
**

**
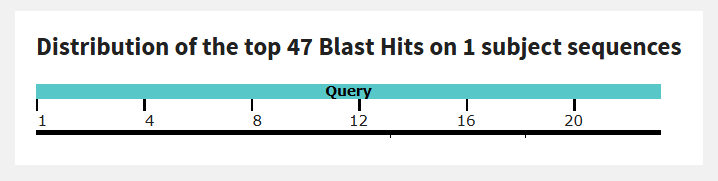
**

**
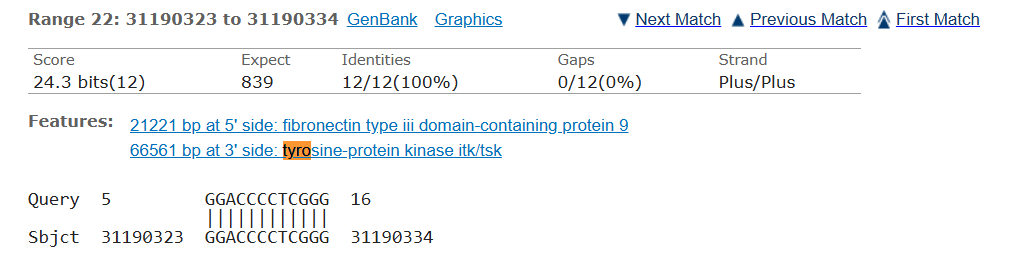
**

**
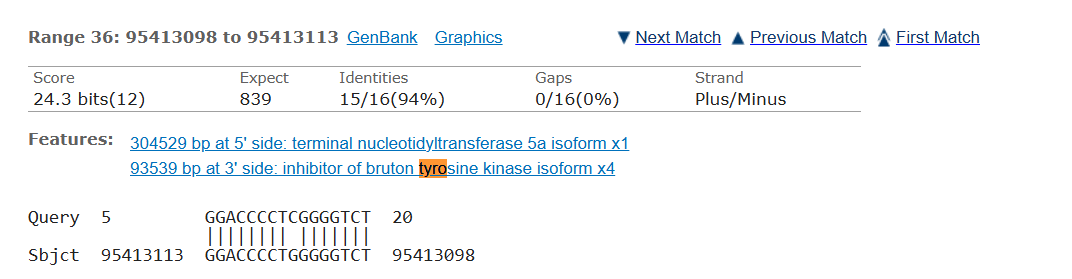
**

**
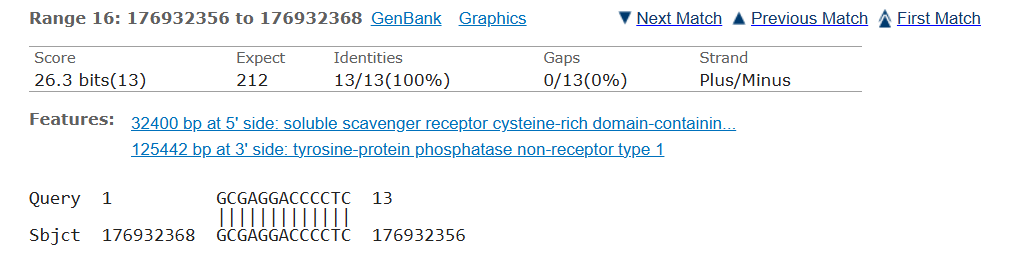
**

Fig. (S8) shows ***mir-661* is located on chr8: 143945191-143945279 [-]. MiR661 sequence (** GCGAGGACCCCUCGGGGUCUGAC**) has been mapped using RAT blast n Rattus norvegicus strain BN/NHsdMcwi chromosome 11, GRCr8 .Sequence ID: NC_086029.1Length: 99753367Number of Matches: 47.**


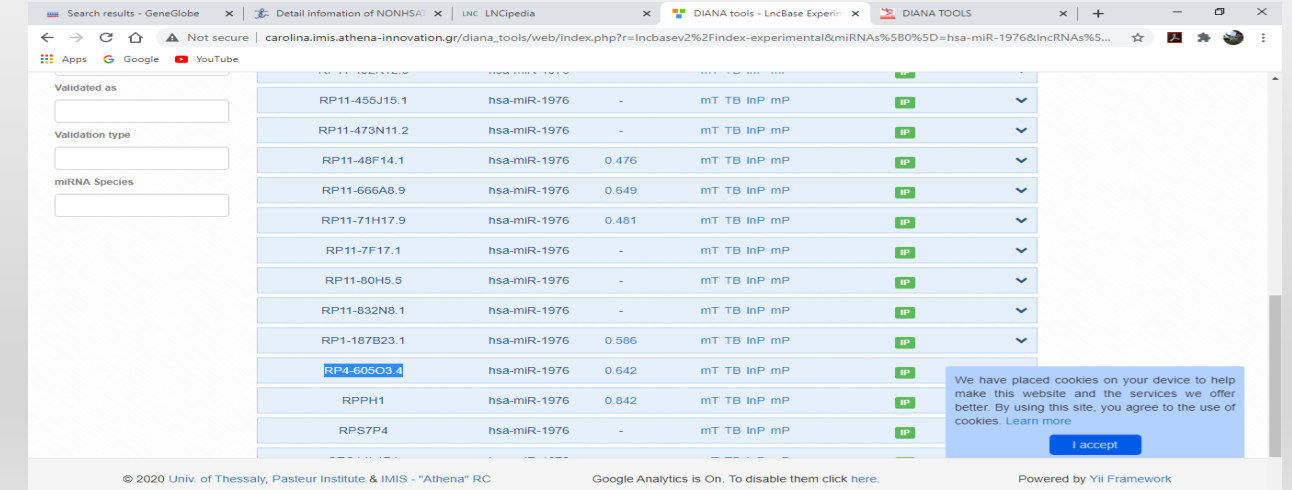


Fig. (S9) shows retrieval of *RP4-605O3.4 lncRNA* from DIANA Tools database

**

Fig. (S10A) shows retrieval of ***RP4-605O3.4 lncRNA*** from **NONCODE** database


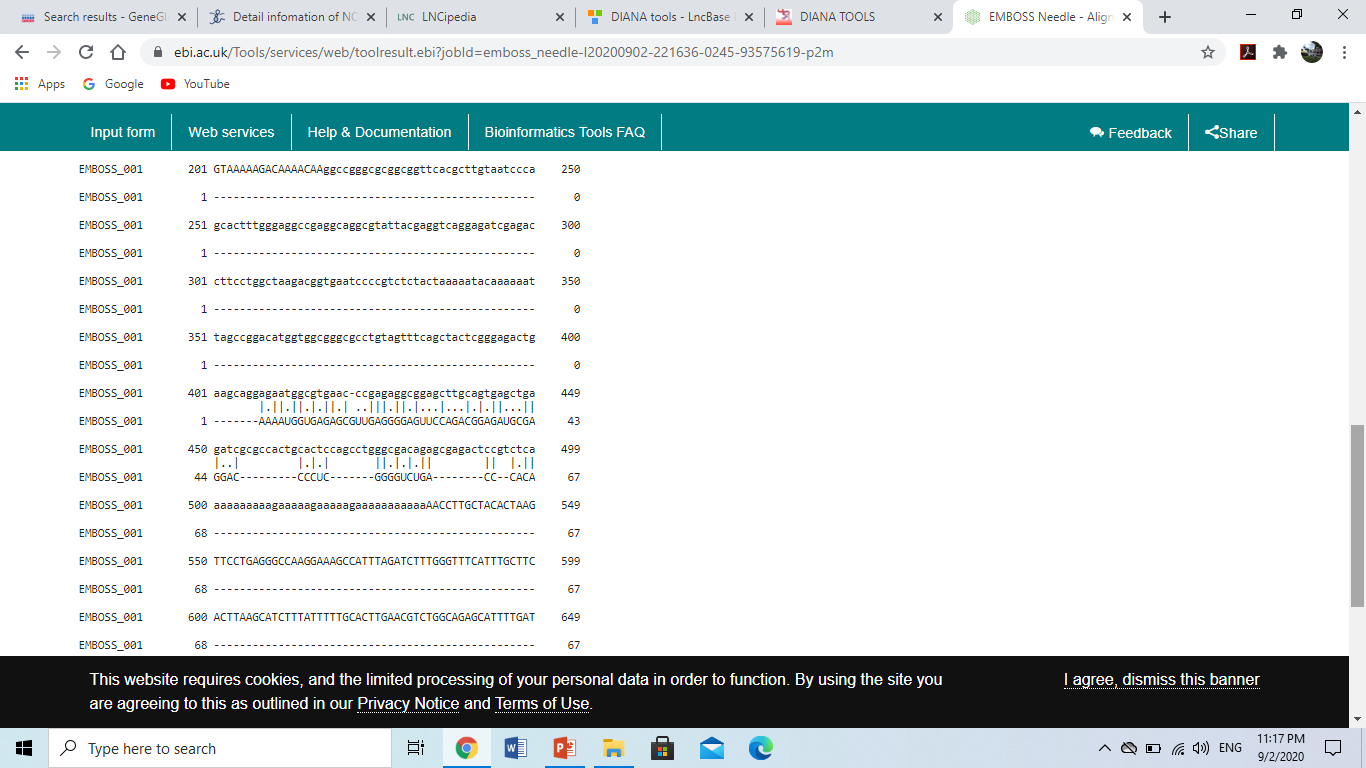


Fig. (S10B) shows alignment between ***miR-611*** and ***lnc-RP4-605O3.4*** using Clustal Omega database

Table (S1): MIQE guidelines checklist:

| **ITEM TO CHECK** | **CHECKLIST** |
| --- | --- |
| **EXPERIMENTAL DESIGN** |  |
| Definition of experimental and control groups | **done** |
| Number within each group | **done** |
| Acknowledgement of authors' contributions | **done** |
| Description | **done** |
| Processing procedure | **done** |
| If frozen - how and how quickly? | **done** |
| Sample storage conditions and duration (especially for FFPE samples) | **done** |
| Procedure and/or instrumentation | **done** |
| Name of kit and details of any modifications | **done** |
| Details of DNase or RNAse treatment | **done (acc.to manufactuer protocol)** |
| Contamination assessment (DNA or RNA) | **done** |
| Nucleic acid quantification | **done** |
| Instrument and method | **done** |
| Purity (A260/A280) | **done** |
| Yield | **done** |
| RNA integrity method/instrument | **done** |
| **REVERSE TRANSCRIPTION** |  |
| Complete reaction conditions | **done** |
| Amount of RNA and reaction volume | **done** |
| Priming oligonucleotide (if using GSP) and concentration | **done** |
| Reverse transcriptase and concentration | **done** |
| Temperature and time | **done** |
| Manufacturer of reagents and catalogue numbers | **done** |
| Storage conditions of cDNA | **done** |
| **qPCR TARGET INFORMATION** |  |
| Sequence accession number | **done** |
| Amplicon length | **done** |
| *In silico* specificity screen (BLAST, etc) |  |
| **qPCR OLIGONUCLEOTIDES** |  |
| Primer sequences | **done** |
| Location and identity of any modifications | done |
| Manufacturer of oligonucleotides | done |
| **qPCR PROTOCOL** |  |
| Complete reaction conditions | **done (acc.to manufactuer protocol)** |
| Reaction volume and amount of cDNA/DNA | **done** |
| Primer, (probe), Mg++ and dNTP concentrations | **done (acc.to manufactuer protocol)** |
| Polymerase identity and concentration | **done (acc.to manufactuer protocol)** |
| Buffer/kit identity and manufacturer | **done (acc.to manufactuer protocol)** |
| Exact chemical constitution of the buffer | **done (acc.to manufactuer protocol)** |
| Additives (SYBR Green I, DMSO, etc.) | **done (acc.to manufactuer protocol)** |
| Complete thermocycling parameters | **done (acc.to manufactuer protocol)** |
| Reaction setup (manual/robotic) | **done (acc.to manufactuer protocol)** |
| Manufacturer of qPCR instrument | **done** |
| **qPCR VALIDATION** |  |
| Specificity (gel, sequence, melt, or digest) | **done** |
| For SYBR Green I, Cq of the NTC | **done** |
| Standard curves with slope and y-intercept | **Done by software** |
| PCR efficiency calculated from slope | **Done by software** |
| r2 of standard curve | **Done by software** |
| Linear dynamic range | **Done by software** |
| Cq variation at lower limit | **Done by software** |
| Evidence for limit of detection | **done** |
| **DATA ANALYSIS** |  |
| qPCR analysis program (source, version) | **done** |
| Cq method determination | **done** |
| Outlier identification and disposition | **done** |
| Results of NTCs | **done** |
| Justification of number and choice of reference genes | **done** |
| Description of normalisation method | **done** |
| Number and concordance of biological replicates | **done** |
| Number and stage (RT or qPCR) of technical replicates | **done** |
| Repeatability (intra-assay variation) | **done** |
| Statistical methods for result significance | **Done** |
| Software (source, version) | **Done** |
